# Genomic regions of durum wheat involved in water productivity

**DOI:** 10.1093/jxb/erad357

**Published:** 2023-09-13

**Authors:** Meryem Zaïm, Miguel Sanchez-Garcia, Bouchra Belkadi, Abdelkarim Filali-Maltouf, Ayed Al Abdallat, Zakaria Kehel, Filippo M Bassi

**Affiliations:** Laboratory of Microbiology and Molecular Biology, Faculty of Sciences, University Mohammed V in Rabat, Morocco; ICARDA, Biodiversity and Integrated Gene Management, P.O. Box 6299, Rabat Institutes, Rabat, Morocco; ICARDA, Biodiversity and Integrated Gene Management, P.O. Box 6299, Rabat Institutes, Rabat, Morocco; Laboratory of Microbiology and Molecular Biology, Faculty of Sciences, University Mohammed V in Rabat, Morocco; Laboratory of Microbiology and Molecular Biology, Faculty of Sciences, University Mohammed V in Rabat, Morocco; Faculty of Agriculture, The University of Jordan, Amman 11942, Jordan; ICARDA, Biodiversity and Integrated Gene Management, P.O. Box 6299, Rabat Institutes, Rabat, Morocco; ICARDA, Biodiversity and Integrated Gene Management, P.O. Box 6299, Rabat Institutes, Rabat, Morocco; New Zealand Institute for Plant and Food Research Limited, New Zealand

**Keywords:** GWAS, moisture stress, QTL, water productivity, wide adaptation, yield stability

## Abstract

Durum wheat is a staple food in the Mediterranean Basin, mostly cultivated under rainfed conditions. As such, the crop is often exposed to moisture stress. Therefore, the identification of genetic factors controlling the capacity of genotypes to convert moisture into grain yield (i.e., water productivity) is quintessential to stabilize production despite climatic variations. A global panel of 384 accessions was tested across 18 Mediterranean environments (in Morocco, Lebanon, and Jordan) representing a vast range of moisture levels. The accessions were assigned to water responsiveness classes, with genotypes ‘Responsive to Low Moisture’ reaching an average +1.5 kg ha^–1^ mm^–1^ yield advantage. Genome wide association studies revealed that six loci explained most of this variation. A second validation panel tested under moisture stress confirmed that carrying the positive allele at three loci on chromosomes 1B, 2A, and 7B generated an average water productivity gain of +2.2 kg ha^–1^ mm^–1^. These three loci were tagged by kompetitive allele specific PCR (KASP) markers, and these were used to screen a third independent validation panel composed of elites tested across moisture stressed sites. The three KASP combined predicted up to 10% of the variation for grain yield at 60% accuracy. These loci are now ready for molecular pyramiding and transfer across cultivars to improve the moisture conversion of durum wheat.

## Teaser

Loci controlling higher water productivity under drought conditions were identified using a strategy involving three distinct germplasm panels.

## Introduction

Durum wheat (2*n*=28, AABB, *Triticum turgidum* L. ssp. *durum*) is a staple and cash crop grown on over 17 million ha worldwide (Tidiane *et al.*, 2019; Xynias *et al.*, 2020). Approximately two-thirds of durum wheat is grown in the Mediterranean Basin, but this area contributes to only half of the worldwide production (Li *et al.*, 2013; Kabbaj *et al.*, 2017). In fact, climate change has and will continue to affect this region, with annual precipitation projected to decrease by 20–40% by the second half of the 21st century (Zittis *et al.*, 2021). Rainfall and temperatures in the Mediterranean dryland areas are largely unpredictable within and between cropping seasons. In past years, North African countries have witnessed a raise in the frequency of drought events, an extension in their length, and an anticipation in their time of occurrence, substantially shifting from late spring to the middle of winter (Belaid *et al.*, 2005; Tramblay *et al.*, 2020; Qi *et al.*, 2022). Since drought stress has a devastating effect on yield and its related traits (Kiliç and Tacettin, 2010; Bilal *et al.*, 2015), North African durum wheat farmers have experienced strong reductions in their productivities. Under such conditions, breeders have committed to the delivery of new varieties with enhanced adaptation mechanisms, by avoiding or tolerating these stresses. Genetic improvement programmes have for a long time attempted to balance the needs of raising overall yield potential, while ensuring cultivars that maintain stable yield performances across seasons. The final productivity of a variety results from the combined effects of genotype (G), environment (E), and their interaction (G×E) (Mohammadi *et al.*, 2015). Thus, the development of superior cultivars requires strategic approaches to combine good stress tolerance with strong yield stability (Mohammadi *et al.*, 2011; Bassi and Sanchez-Garcia, 2017).

Yield stability refers to the ability of certain genotype to ensure good yield performances despite the fluctuations of growing conditions occurring across environments, and it is normally associated with the G×E component. Several decades of studies have demonstrated that stability is controlled by genetic factors interacting with the environment. As such, it is possible to improve the stability of a genotype via pyramiding multiple positive alleles for this trait. Breeders approach this need by testing the genotypes under a vast range of environments and seasons, to then derive what are defined as stability scores (Malosetti *et al.*, 2013) and then use these to identify stable genotypes across environments. One such score widely used in durum wheat breeding is the AMMI wide adaptation index (AWAI) score that utilizes the additive main effects and multiplicative interaction (AMMI) capacity to partition the G×E into sub-factors, to then estimate a weighted value to be assigned to the genotype (Bassi and Sanchez-Garcia, 2017). However, a stable variety can also be obtained by pyramiding multiple positive alleles at loci controlling discrete interactions with the environment. For instance, a drought tolerant variety would be able to maintain its yield performance (i.e., stability) even when moisture stress occurs. The concept of water productivity (WP) is linked to yield stability and potential as it has been used in plant breeding to define genotypes capable of using moisture in a more efficient way, and hence achieve higher productivity at the same level of moisture input (Anyia *et al.*, 2008). Although the application of this concept was originally proposed to define the response of genotypes to increasing irrigation rates, it has become even more important to assess the response to moisture stress, when water availability is particularly scarce (Bhouri Khila *et al.*, 2021). Wheat’s most sensitive growth stages to water stress are mainly stem elongation and booting, followed by anthesis and grain filling (Blum and Pnuel, 1990; Shpiler and Blum, 1990; del Moral *et al.*, 2003). Water deficit around anthesis may lead to a loss in yield by reducing spike and spikelet number and the fertility of surviving spikelets, whereas water deficit during the grain-filling period reduces grain weight (Karam *et al.*, 2009). Geerts and Raes (2009) indicate that scarce moisture can increase WP for various crops without causing severe yield reductions. Zhang *et al.* (2006) demonstrated that under rainfed conditions, wheat grain yield, harvest index, and WP were greatly improved under regulated deficit irrigation when compared to the non-water stressed treatment. Maximizing WP may be economically more profitable for the farmer than maximizing yields or land productivity (English, 1990) in areas where water is the most limiting factor. The results of Karrou and Oweis (2012) showed that in general a reduced irrigation of one-third of full supplemental irrigation gave the highest rate of increase in grain yield and WP. Grain yield reductions due to the application of two-thirds supplemental irrigation were around 10% on average, whereas differences in total WP of crops grown under full irrigation compared to deficit irrigation were not significant.

Beyond the application of stability systems to adapt to all conditions, there are several discrete traits that have been proposed as favouring the adaptation of durum wheat to moisture stress. A simplified list of these would include early maturity to avoid terminal stress (Gupta *et al.*, 2020), good coverage of ground to favour shading and prevent moisture transpiration from the soil (Yadvinder *et al.*, 2014), a root system architecture more suitable to access the residual moisture in the different soil layers (Yadvinder *et al.*, 2014; Lilley and Kirkegaard, 2016; El Hassouni *et al.*, 2019), and improvement of specific yield components, with a particular attention to grain size (Mohammadi *et al.*, 2019). In that sense, breeders seek to identify and pyramid these traits to achieve better stability when moisture stress occurs (Araus *et al.*, 2008; Reynolds *et al.*, 2009; Tuberosa, 2012; Sukumaran *et al.*, 2018). Therefore, the knowledge of genetics and gene action of these traits is essential for generating stable varieties (Habash *et al.*, 2009). Molecular markers technology offers the possibility to identify and track these positive alleles (Collard and Mackill, 2008; Ceccarelli, 2015). Genome wide association study (GWAS) is an approach that helps determine significant relationships between the allelic make up (i.e., haplotypes) of a genotype and its field response. Such an approach was used for the identification of novel quantitative trait loci (QTL) with potential implications for durum wheat breeding programmes, such as loci associated with variation in kernel size (Fiedler *et al.*, 2017), grain yield and its components (Mangini *et al.*, 2018; Sukumaran *et al.*, 2018; Wang *et al.*, 2019), but also response to moisture changes and roots. Maccaferri *et al.* (2011) used association mapping to dissect the genetic basis of drought-adaptive traits and grain yield (GY) in a collection of 189 elite durum wheat accessions evaluated in 15 environments with differing water availability during the crop cycle (from 146 to 711 mm). For GY, significant associations were mostly detected in one environment only, while decreasing rapidly from two to five environments and with only one marker found significant in six environments. In another study, Maccaferri *et al.* (2016) used linkage and association mapping for root system architecture in two recombinant inbred line populations and one association mapping panel of 183 elite durum wheat accessions evaluated as seedlings revealed 20 clusters of QTL for root length and number, as well as 30 QTL for root growth angle (RGA). Divergent RGA phenotypes observed by seminal root screening were validated by root phenotyping of field-grown adult plants.

In the present study, we aimed at broadening the understanding of the genetic factors involved in controlling WP and moisture stress adaptation in durum wheat. Therefore, we investigated the performances of a large ‘discovery panel’ of durum wheat accessions across 18 environments experiencing different degrees of in season moisture. Beyond the identification of stable and top performing entries, this investigation sought to define discrete clusters of WP types. GWAS was then used on the ‘discovery panel’ to identify haplotypes more frequently present in the most water responsive genotypes, which were then investigated in a second ‘confirmation panel’. Finally, to ensure these critical loci can be readily incorporated into novel cultivars, kompetitive allele specific PCR (KASP) markers were developed to tag them and then confirmed for their ability to predict moisture stress adaptation on a third ‘validation panel’.

## Materials and methods

### Plant material

This study evaluated three discrete germplasm panels and all associated phenotypic and genotypic data are made available as Supplementary Table S1. The first panel is defined as the ‘discovery panel’ and it includes 384 durum wheat entries including landraces, elites, and cultivars (Supplementary Table S2). The kinship of this panel was previously presented by Kabbaj *et al.* (2017), and it has already been used to identify the genomic loci involved in resistance to a damaging insect pest (Bassi *et al.*, 2019), phenology (Gupta *et al.*, 2020), and response to heat stress (El Hassouni *et al.*, 2019). This panel was tested in its entirety at some environments, whereas a subset was used in other environments as explained in more detail below. The second set of entries is defined as the ‘confirmation panel’ and it includes 80 ICARDA’s elites that constituted the 2019 international nurseries 42nd International Durum Observatory Nursery (IDON; Supplementary Table S3). The third set is defined as the ‘validation panel’ and it includes 80 ICARDA’s elites that constituted the 2020 international nurseries 43rd IDON (Supplementary Table S4). The last two panels share some co-ancestry as can be expected from germplasm developed by a breeding programme. The ‘discovery’ panel also includes few entries that were later used as parents to derive the subsequent two panels.

### Field trials and management

**Fig. 1.**
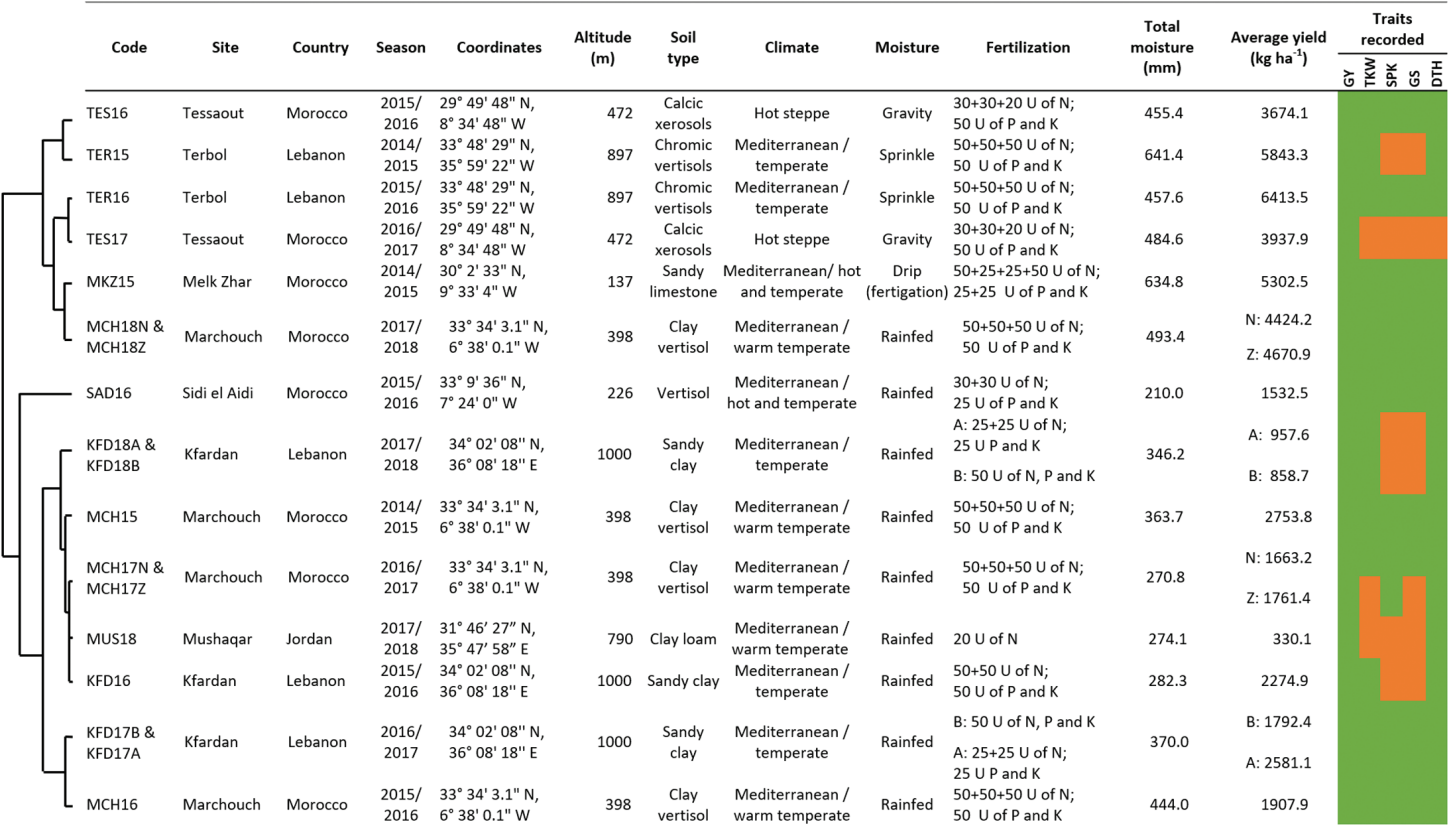
Description of the testing environments used for the ‘discovery panel’, and their PCA hierarchical clustering based on climatic factors. GY: Grain yield (kg ha^-1^), TKW: 1000 kernel weight (g), SPK: Spike density per m^2^, GpS: Grain per spike, PLH: Plant height (cm), DTH: Days to heading.

The ‘discovery panel’ was assessed during the 2014–15, 2015–16, 2016–17, and 2017–18 growing seasons in 18 contrasting environments as described in Fig. 1. Four were in Morocco: Marchouch (MCH), Sidi el Aydi (SAD), Melk Zhar (MKZ), and Tessaout (TES); two in Lebanon: Terbol (TER) and Kfardan (KFD); and one in Jordan: Musghar (MUS). The experimental design was an augmented design with four replicated checks in the 2014–15 (15) and 2015–16 (16) growing seasons in MCH15, MCH16, SAD16, MKZ15, MKZ16, TES16, TER15, TER16, KFD16, and MUS18 during 2017–18 season (18). During the 2016–17 (17) and 2017–18 (18) seasons, a subset of 144 genotypes was selected and used to run an alpha-lattice design with two replications and 12 incomplete blocks, at MCH17, MCH18, SAD17, TES17, KFD17, and KFD18. Each entry was planted in plots of six rows of 5 m in length, and row spacing was 0.2 m, for a total sown surface of 6 m^2^ at a seeding rate of 120 kg ha^–1^. Agronomic practices follow a timely sowing date between 15 November and 15 December with a base pre-sowing fertilizer application of 50 kg ha^–1^ of N, P, and K. Planting occurred after a legume crop season. During the 2016–17 and 2017–18 seasons in MCH, two management conditions were used: normal sowing (MCHN) following standard land preparations and tillage, and zero tillage (MCHZ) on a fully retained faba bean stubble. Both sowings were conducted using the same seeder, even though it was specifically developed for zero till practices. At stage 14 of the Zadok’s scale (Z) herbicide was applied in a tank mixture to provide protection against both monocots and dicots. A week after herbicide application, ammonium nitrate was provided to add 36 kg ha^–1^ of N. When in season moisture exceeded 350 mm a final application of urea was used at flowering to deliver an additional 46 kg ha^–1^ of N. In KFD17 and KFD18, two kinds of fertilizer applications were made: KFDA with only basal fertilization (50 kg ha^–1^ of N, P, and K) and KFDB with an additional 50 kg ha^–1^ of urea at Z15. In MKZ, the first basal fertilization was followed by five split applications each of 20 kg ha^–1^ of N via fertigation through drip pipes. Three sites were irrigated: TES, where four gravity irrigations of 35 mm each were provided after Z10, Z18, Z45, and Z65; MKZ, where 12 irrigations of 10 mm each were provided via drip irrigation at 1 week intervals, initiating 2 weeks after Z10 to Z89, and TER, where two sprinkle supplemental irrigation of 20 mm each were provided before Z10 and after Z65. The remaining experiments were conducted under rainfed conditions with total rainfall values and other details presented in Fig. 1.

The station of Sidi el Aydi (SAD) in Morocco was identified by the global initiative CRP WHEAT as an ideal site to test for drought tolerance of wheat, and for this reason it was also used to screen the two other panels. The ‘confirmation panel’ was tested at SAD and MCH, whereas the ‘validation panel’ was tested just at SAD. Both panels were planted following an augmented design with four replicated checks, during the seasons 2018–19 and 2019–2020, respectively. The total moisture recorded during 2018–19 was 296 and 154.6 mm at SAD and MCH, respectively, whereas during 2019–2020 at SAD was 286 mm, which constituted strong moisture stress for durum wheat.

### Phenotyping

Days to heading (DTH) was recorded as days elapsing between sowing and 50% of plants showing emerging heads. At maturity, the number of fertile spikes were counted in 0.25 m^2^ and this value was multiplied by four to derive the number of spikes per m^2^ (SPK). Grain yield (GY, kg ha^–1^) was recorded by harvesting the central four rows of each plot, weighting it on a precision scale and dividing this value by the plot surface. From the harvest of each plot, 1000-kernel weight (TKW, g) was determined by counting 500 randomly selected grains on a ‘Chopin Numigral’ counter followed by weighting on a precision scale. The number of grains per metre square (Gr.m^–2^) was calculated using the total weight of the plot, divided by the harvested surface and the estimated weight of one kernel, as per (1):


$$\begin{array}{l} Gr.m^{-2}=\frac{Harvested\;weight\;of\;plot}{plot\;area\;x\frac{TKW}{1000}} \end{array}$$ (1)

The number of grains per spike (GpS) was then derived by dividing the number of grains per metre square (1) by the number of spikes recorded for the same area as follows (2):


$$\begin{array}{l} GpS=\frac{Gr.m^{-2}}{Spk.m^{-2}} \end{array}$$ (2)

As detailed in Fig. 1, GY was recorded in all environments, whereas the other traits were collected only in some environments.

### Field data analysis

Analysis of variance (ANOVA) was performed using Genstat for the augmented designs, while alpha lattice and the combined analysis were run on GEA-R 4.1 in the R environment (Pacheco *et al.*, 2015). Combined ANOVA across mega-environments was obtained by linear model fitted considering genotypes as a fixed term (R Core Team, 2017). Best linear unbiased estimates (BLUEs) were calculated for each genotype in each environment defining genotypes as fixed effects using the R package ASReml-R (Butler *et al.*, 2009). The package ASReml-R was also used to estimate the narrow-sense heritability. Broad-sense heritability was calculated separately for each design by Genotype × Environment Analysis with R (GEA-R) version 4.1. The ratio of variance accounted by each source of variations (G, E, and G×E) was calculated by dividing the sum of the square of each source by the total sum of the square.

For grain yield, G×E was partitioned by the AMMI model using the R package Agricolae (De Mendiburu and Yaseen, 2020). The ‘AMMI wide adaptation index’ (AWAI) measures the distance of each genotype from each significant axis and it was calculated using the following formula, presented by Bassi and Sanchez-Garcia (2017):


$$\begin{array}{l} AWAI=\sum_{i}s_{i}\;\times \;|PC_{i}| \end{array}$$

where *i* is the number of significant IPCs determined by the classical F-test in R (R Core Team, 2017), *s*_*i*_ is the percentage of total G×E variance explained by each IPC, and PC is the actual IPC value. AWAI values close to ‘0’ are obtained for the most widely adapted and stable germplasm (Malosetti *et al.*, 2013). As indicated by Bassi and Sanchez-Garcia (2017), a biplot between the genetic (G) component of yield (i.e. yield potential) and the interaction (G×E) component (i.e. yield stability) was used to determine the best genotypes combining both G and G×E for grain yield. The AWAI index explaining G×E was presented as ratio to minimum value, and values close to ‘1’ were obtained for the most widely adapted and stable genotypes. To define the genetic component of GY obtained across environments with vast differences in the average performances, the actual values were converted to a ratio of the top performing entry at each environment and then averaged across.

**Fig. 2.**
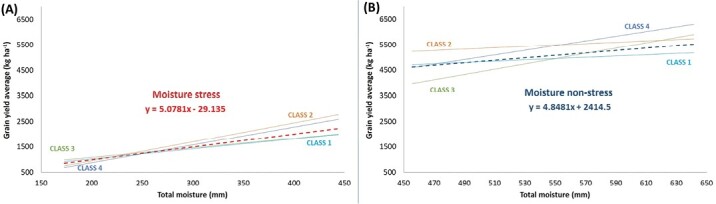
Trendlines of linear regression representing water productivity as a biplot of the total moisture in each environment against the grain yield at that environment. One representative genotype example for each water productivity class (Table 1) is presented and compared to the average performance of all genotypes in each environment (dashed line). The trendline colour cyan represent the ‘class 1’, the orange for ‘class 2’, green for ‘class 3’, and blue for ‘class 4’, Moisture stressed environments are presented in (A) and non-moisture stressed in (B).

A climate matrix was developed for each environment, splitting the records into five growth stages: 1 month before sowing, sowing until the end of the vegetative stage, flowering stage, grain-filling period, and physiological maturity period. Simple linear regression was conducted between the climatic matrix and the response of genotypes at each site for GY. The climatic factors having a significant effect (*P*<0.05) were used to perform hierarchical (Fig. 2).clustering among environments using the R package FactoMineR (Josse *et al.*, 2008).

### Assignment of genotypes to water productivity classes

The water productivity (WP) is calculated using the following formula:


$$\begin{array}{l} WP=\frac{Grain\;yield}{Total\;moisture} \end{array}$$

To define the average trend, the average GY performances of each environment was plotted against the total moisture of that environment, which corresponds to a graphical representation of the average WP trend. To increase the accuracy of this trend, the environments were split into two groups, one defined as ‘stressed’ including 11 environments experiencing moisture stress, and the second defined as ‘non-moisture stressed’ including seven environments where moisture stress did not occur. The slope (b) of WP was calculated for each group, reaching 5.08 for the moisture stress cluster and 4.85 for non-moisture stress. These values represent then the hypothetical average performance of a given genotype tested at that group of clusters. Hence, higher values (steeper response to water increase) would be obtained by genotypes with higher WP, whereas lower values (flatter curve) would be associated with less responsive genotypes. To assess this, the GY performance of each genotype at each environment was plotted against the moisture level of that environment and the actual slope value (b_i_) for the two trendlines (moisture stressed and non-moisture stressed) were calculated (Fig. 2). Based on these b_i_ values, genotypes were assigned to four different water responsive classes representing more or less responsiveness compared to the average trend (Table 1). However, to ensure that the trendline explained the observed changes in moisture, genotypes for which the regression value between GY and moisture levels was not significant (*P*<0.01) were assigned to a fifth class of water unresponsive behaviour.

**Table 1.** Water productivity classes

Classes	Definition	r^2^	b_i_^*c*^ stressed	b_i_^*c*^ non-moisture stressed
1	Stable water response	^**a^	b_i_<5.08	b_i_<4.85
2	Responsive to low moisture	^**^	b_i_>5.08	b_i_<4.85
3	Responsive to high moisture	^**^	b_i_<5.08	b_i_>4.85
4	Highly water responsive	^**^	b_i_>5.08	b_i_>4.85
5	No water response	ns^*b*^	.	.

^*a* **^ *P*<0.01.

^*b*^ ns, not significant.

^*c*^ b_i_ actual slope value of water productivity

### Genotyping and association mapping analysis

The ‘discovery panel’ was genotyped with a 35K Affymetrix Axiom wheat breeders array to generate 7652 polymorphic single nucleotide polymorphisms (SNPs) with 98 to 100% identity when blast aligned to the Svevo genome (Maccaferri *et al.*, 2019), less than 1% missing data, minor allele frequency higher than 5%, and heterozygosity less than 5% as detailed in Kabbaj *et al.* (2017). These authors also defined a kinship structure of 10 sub-clusters. Genome wide linkage disequilibrium (LD) decay analysis were performed by Bassi *et al.* (2019) and defined as 51.3 Mbp. GWAS was performed for the panel using as phenotypic input the BLUEs of each trait at each environment, and the combined analysis of the two moisture groups (stressed and non-moisture stressed). TASSEL 5 software (Bradbury *et al.*, 2007) was used for the analysis imposing DTH as a covariate to avoid identifying flowering genes, since these were already described in Gupta *et al.* (2020). Two models were used and compared using two additional covariate parameters: Q (population structure) and K (Kinship). The Q model was performed using a general linear model (GLM), and the Q + K model using a mixed linear model (MLM). The best model for each trait was selected based on quantile-quantile (Q-Q) plots (Sukumaran *et al.*, 2012). Significant marker-trait associations (MTA) were determined using a Bonferroni correction by LD as suggested by Duggal and Beaty (2008) for *P*<0.05 corresponding respectively to a LOD=2.69 (Bassi *et al.*, 2019). In addition, Pearson’s critical values (Pearson, 1895) for the correlation r was squared to obtain a critical r^2^ of 0.024 (*P*<0.01) and used to determine significant markers explaining sufficient ratio of the total phenotypic variation. Any marker-trait associations (MTAs) with LOD and r^2^ superior to these cut-offs were considered valid and presented here. MTAs falling at a distance inferior to twice the LD (102.6 Mbp) were deemed to be too physically close to be resolved by this panel into distinct loci and hence were assigned the same QTL identifier. QTL were defined as ‘consistent’ when it included MTAs significant in both the combined analysis across sites, and in more than one individual environment.

The ‘confirmation panel’ was genotyped using a 23K array chip developed by SGS - Institut Fresenius TraitGenetics Section (Germany) which incorporates 14.5K SNPs from the 90K Infinium Array, 8.5K SNPs from the Axiom Array, and 265 SNPs reported as linked to genes in the literature (Vitale *et al.*, 2021). Marker curation was conducted as for the ‘discovery panel’, resulting in 6325 polymorphic SNPs. These were also aligned to the Svevo genome assembly (Maccaferri *et al.*, 2019). A kinship structure of eight sub-clusters was identified (Supplementary Table S3) and linkage analysis revealed that the LD was 21.2 Mbp (Supplementary Fig. S1) which resulted in a significant r^2^=0.05 (*P*<0.01). The two genotyping platforms were merged using the Svevo genome assembly as a scaffold based on physical overlap. Similar to the ‘discovery panel’, GWAS was conducted using flowering time as a covariate. Significant MTAs were determined using Bonferroni correction for *P*<0.05 corresponding to LOD=4.1. ShinyCircos software (Yu *et al.*, 2018) was used to graphically represent the MTAs and QTL identified by both ‘discovery’ and ‘confirmation’ panels. Only those ‘consistent’ QTL identified by GWAS in the ‘discovery panel’ and then also identified by GWAS in the ‘confirmation panel’ were deemed ‘true’ positives and studied further.

The most representative marker for each ‘true’ QTL was selected as the one having the highest LOD and r^2^ within the QTL interval. Discrete classes of genotypes from the ‘confirmation panel’ were then defined based on their allelic combinations at these representative markers. These classes were defined as ‘haplotypes’. The phenotypic performances of the ‘confirmation panel’ genotypes belonging to each haplotype class were defined as a random effect, and a linear model was run to determine significance difference by the least significant difference (LSD) using the LSD.test function of the *agricolae* package (R Core Team, 2017; De Mendiburu and Yaseen, 2020).

The 35K or 25K array probe sequences underlying the most interesting QTL were submitted to Laboratory of the Government Chemist (LGC, UK registration 2991879) to run their proprietary software to assess their suitability to design KASP primers. For each QTL, four potential primer sets were synthetized and run on the ‘validation panel’. For each KASP marker that amplified and showed polymorphism, its allelic score was regressed against the GY value and a significance threshold was set at r^2^>0.053 (*P*<0.01). In addition, the top 20 yielding genotypes were defined as the ‘positive’ cases and the worst 20 genotypes as the ‘negative’ cases. The marker score was then evaluated among the positive and negative cases to define correct SNP calls (true positive or true negative) and incorrect SNP calls (false positive and false negative). The marker accuracy was then calculated as the ratio of the correct allelic call among all, sensitivity as the ratio of the correct positive allelic calls among all, and specificity as the ratio of the correct negative allelic calls among all. The primer sequence of the markers is protected by commercial rights and cannot be disclosed here, but these can be purchased by all users as a service via LGC indicating the marker names provided.

## Results

### Phenotypic variation under moisture stressed and non-moisture stressed environments

Analysis of variance revealed significant differences (*P*<0.01) for the genotype (G), environment (E), and their interaction (G×E) for most of the traits (Supplementary Table S5). The E effect explained most of the variation for GY (73%), GpS (69.3%), TKW (83%), SPK (88%), and DTH (84%), whereas the G effect explained the largest variation for GpS (16%). The G×E interaction showed a larger contribution to the total variability compared to the G effect for GY and SPK. Good heritability was obtained in all environments for all traits.

Vast phenotypic variation was recorded for all traits across the 18 environments (Supplementary Fig. S2). The highest average GY was recorded in TER16 (6413.5 kg ha^–1^), whereas MUS18 had the lowest average GY (330.1 kg ha^–1^) (Fig. 1). Moisture data shows patterns of variation across environments, with some sites having a prevalence of drought events (MUS, KFD, SAD, MCH; Fig. 1). GY performances were significantly (*P*<0.05) influenced by the total water input during vegetative, flowering, and grain-filling stages, and the maximum temperature during the flowering stage (Supplementary Table S6). Because of their significant effect on yield, these climatic factors were used to cluster the environments by principal component analysis (PCA) in two mega-environments: (i) moisture stressed (MUS18, KFD17, SAD16, MCH15, MCH17, KFD16, MCH16, and KFD18) and (ii) non-moisture stressed (TES16, TER15, MKZ15, TES17, TER16, and MCH18).

To avoid range effects, grain yield (BLUE) was converted to ‘ratio to the max’, to scale the variation based on the best performing entry. Under moisture stressed conditions the CIMMYT line GID: 800032262 (3041 kg ha^–1^) was the top yielding. Among the top highly performing entries, the ICARDA wide crosses GID:800032191 (2564 kg ha^–1^), GID:800043267 (2375 kg ha^–1^), GID: 800032178 (2242 kg ha^–1^) and the elite lines GID: 800032351 (2204 kg ha^–1^) and GID: 800030179 (2191 kg ha^–1^). The top yielding line under non-moisture stressed conditions was the ICARDA elite GID: 800043103 (7517 kg ha^–1^); the Moroccan line GID: 4984522 (6407 kg ha^–1^) was also among the high yielding.

**Fig. 3.**
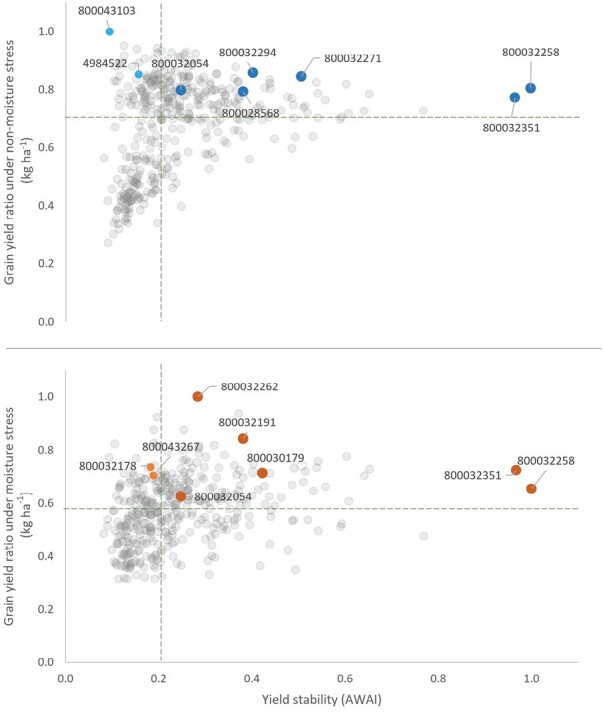
AMMI wide adaptation index (AWAI) referring to yield stability against the ratio to the max of yield potential across 11 moisture-stressed (upper) and seven non-moisture stressed (lower) environments. Dashed lines trace the average for each axis.

Partitioning the G×E effect by AMMI defined 17 significant principal components (PCs), of which the first three combined accounted for 67.9% of the variation. The definition of the AWAI score determined an average performance equal to 0.2, with the two most stable lines being GID: 800032258 (a CIMMYT elite line) and GID: 800032351 (an ICARDA elite line). The bi-plot combining GY performances across sites and stability (AWAI) provides an ideal selection index to combine G and G×E effects (Fig. 3) (Bassi and Sanchez-Garcia, 2017). Combined analysis under moisture stress identified 24% of genotypes achieving GY and AWAI values above the average of the tested lines. Lines GID: 800032262, GID: 800032261, and GID: 800032280 were the top three stable and yielding lines. The ICARDA elite line GID: 800030179 and GID: 800032191 were also among the highly performing. While under non-stress conditions, 32% of the tested entries had higher yield and AWAI than the average. The high yielding genotypes GID: 800043103 and GID: 4984522 were not stable, while the Australian elite line GID: 800032336, CIMMYT line GID: 800032267 and the ICARDA line GID: 800032342 combined high yield with good stability.

Beside stability per se, several traits contribute to the adaptation of genotypes to the environment. To determine which traits contributed to GY variation, correlation analysis was performed for each individual and mega-environment. This interaction (Table 2) revealed that GpS influenced (*P*<0.001) GY in all environments. TKW had an effect only in five out of nine moisture-stressed environments and in all the non-moisture stressed ones. Overall, SPK was not significantly correlated with GY under non-moisture stress, only two environments showed a highly significant relationship, while it accounted for 60% of yield variation in moisture stressed environments. In most of the environments flowering time showed a highly significant correlation with GY.

**Table 2.** Correlation analysis for all traits against grain yield across moisture and non-moisture stressed conditions

Mega-environment	Environments	Moisture (mm)	DTH	TKW	SPK	GpS
Moisture stressed	SAD16	210.0	•^*a*^	•	ns^*b*^	•
	MUS18	247.0	•	- ^*c*^	-	-
	MCH17N	270.8	•	ns	ns	•
	MCH17Z	270.8	•	-	ns	-
	KFD16	282.3	•	•	-	-
	KFD18A	346.2	ns	ns	-	-
	KFD18B	346.2	ns	ns	-	-
	MCH15	363.7	•	•	•	•
	KFD17A	370.0	•	ns	•	•
	KFD17B	370.0	ns	ns	•	•
	MCH16	444.0	•	•	•	•
	Average	320.1	•	•	•	•
Non-moisture stressed	TES16	455.4	ns	•	•	•
	TER16	457.6	•	•	ns	•
	TES17	484.6	-	-	-	-
	MCH18N	493.4	•	•	ns	•
	MCH18Z	493.4	•	•	•	•
	MKZ15	634.8	•	•	ns	•
	TER15	641.4	•	•	-	-
	Average	522.9	•	•	ns	•

^*a*^ Significant at the probability level 0.001 (•).

^*b*^ ns: not significant.

^*c*^ ‘-’ Not available.

### Water productivity performance of genotypes

Climatic regression against GY identified that moisture amount during the vegetative, flowering, and grain-filling stages were the most significant climatic factors, explaining more than 70% of the variation (Supplementary Table S6). To better elucidate the relationship between GY and moisture, a WP value was calculated for each genotype. The average WP was estimated at 7.2 kg ha^–1^ mm^–1^ showing a significant linear relationship (r^2^=0.327) to the increase in moisture levels, resulting in an average WP of 5.1 kg ha^–1^ mm^–1^ across moisture stressed environments, whereas it reached 9.7 kg ha^–1^ mm^-1^ across non-moisture stressed environments (Supplementary Fig. S3).

A subset of 120 genotypes, that have been assessed in all environments, were assigned to WP classes based on their respective trend of yield variations plotted against the moisture levels across environments. Twenty-five percent of the tested entries were assigned to class 3 ‘Responsive to high moisture’ and 25% to class 4 ‘Highly water responsive’, whereas class 2 ‘Responsive to low moisture’ incorporated 20% of genotypes, 17% belonged to class 5 ‘No water response’, and 13% to class 1 ‘Stable water response’. From a breeding perspective, classes 2, 3, and 4 are the most interesting because they identify genotypes capable of producing more yield per water input compared to the average. Interestingly, genotypes GID: 800030179, GID:800043267, and GID: 800032178 resulted among the highly WP performing elite lines under moisture stress, belonging to class 2 ‘Responsive to low moisture’. While under non moisture stress, the ICARDA elite line GID: 800028568 belonging to class 3 ‘Responsive to high moisture’ was among the highest for WP. Genotype GID: 800032054, a CIMMYT line with top yield under both moisture conditions, belonged to class 4 ‘Highly water responsive’ (Supplementary Table S7).

### QTL controlling traits under moisture stressed and non-moisture stressed conditions

**Fig. 4.**
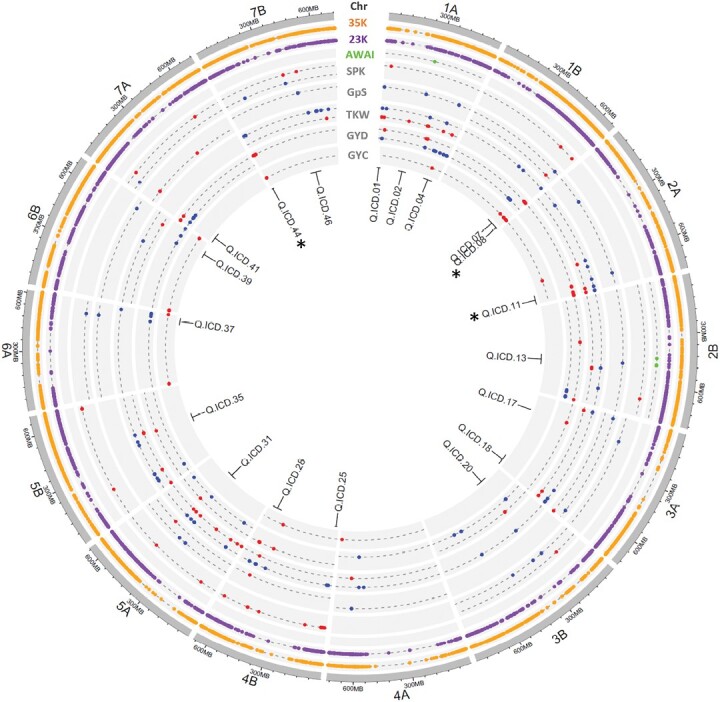
Circos displays QTL associations in durum wheat genome across panels, revealing genetic markers linked to traits and responses to moisture stress. The outermost circle shows the Svevo durum wheat genome assembly (Macaferri *et al*. 2019), including its chromosomes, followed by the distribution of the 35K Axiom polymorphic probes in the ‘discovery panel’ (35K) and the 23K SNP probes in the ‘confirmation panel’ (23K). The following tracks represent results of significant marker trait association (MTA) identified in the ‘discovery panel’ for AMMI wide adaptation index (AWAI) coded as green dots, spike density per m^2^ (SPK) and grain per spike (GpS), 1000 kernel weight (TKW), and grain yield (GYD) across individual and combined environments. The following tracks represent MTA for GY identified in the ‘confirmation panel’ (GYC). The MTA identified in moisture stressed environments are colour coded as red dots, whereas those identified in non-moisture stressed conditions are coded as blue dots. The innermost circle provides the QTL labels for reference. Asterisks represent the QTL confirmed by the GYC.

Initially, GWAS was conducted on the ‘discovery panel’, involving individuals and both combined mega-environments analyses for all traits, resulting in the identification of 280 significant MTAs. The MTAs explained from 3% to 22% of the phenotypic variation and the LOD ranged from 2.7 to 7.2. MTAs which were distributed across 47 discrete QTL (Fig. 4; Supplementary Table S8).

Under non-moisture stress environments, four consistent QTL (Q.ICD.04, Q.ICD.07, Q.ICD.37, and Q.ICD.39) associated with GY were identified on chromosomes 1A, 1B, 6A, and 6B (Fig. 4). Q.ICD.37 was also associated with GpS and SPK, whereas Q.ICD.39 and Q.ICD.04 also controlled TKW and GpS.

Under moisture stressed conditions, GY was associated with 14 loci (Fig. 4). Among these, Q.ICD.08, Q.ICD.11, Q.ICD.17, Q.ICD.20, Q.ICD.28, and Q.ICD.44 on chromosomes 1B, 2A, 3A, 3B, 4B, and 7B, respectively, were identified in two or more stressed environments. Interestingly, locus Q.ICD.28 was also associated with TKW, SPK, and GpS, whereas Q.ICD.44 controlled TKW, in addition to GY.

A comparison of significant loci for GY across stressed and non-moisture stressed conditions identified a consistent locus on chromosome 7A (Q.ICD.41), also controlling GpS and SPK, on chromosome 5A (Q.ICD.31) associated with GY, TKW, and SPK, and on chromosome 1A (Q.ICD.01) controlling GY, TKW, SPK, and GpS. This last QTL was the most frequently identified region across all environments.

GWAS conducted for yield stability (AWAI) revealed two QTL (Q.ICD.02 and Q.ICD.13) on chromosomes 1A and 2B. Interestingly, both QTL were linked to GpS and TKW. In addition, for TKW, two additional loci (Q.ICD.18 and Q.ICD.35) not associated with GY were identified on chromosomes 3A and 5B.

Conducting GWAS for the ‘confirmation panel’ tested at SAD and MCH, confirmed the importance of Q.ICD.08, Q.ICD.11, and Q.ICD.44, which were also identified as important QTL in the ‘discovery panel’ under moisture stress conditions.

### Effect of different allele combination on water productivity classes

**Fig. 5.**
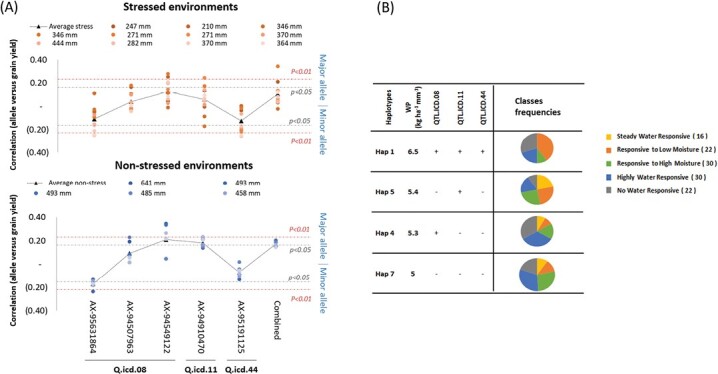
Allele-grain yield correlation and haplotype frequencies in diverse environments. (A) Correlation between allelic call of five major markers representing three QTL and grain yield at 18 environments, presented for the moisture stressed and non-moisture stressed separately. Each environment is named as the total of its moisture, and colour coded from darker to brighter orange for severity of drought, and darker to lighter blue for decreasing moisture content. The average performance is presented as a black triangle. The Pearson’s significant cut off are presented for both major and minor alleles. (B) Allelic haplotype effects of the significant loci on water productivity of 120 genotypes grown under 18 environments. Left: The accessions were divided into four groups based on their haplotype for three major QTL: ‘+’ mark the positive and ‘-’ the negative alleles. Right: The haplotype frequencies of each water response class.

To determine the allelic effect on grain yield across environments, the main representative marker of each QTL was investigated as single marker regression at all locations (Fig. 5). The major allele of AX-94549122 was strongly correlated with grain yield under both moisture conditions, whereas AX-95631864 was the minor allele that was associated with both conditions. Hence, these two loci contributed to yield overall. The AX-94910470 major allele is most important for non-moisture stressed environments, whereas the AX-95191125 minor allele is linked only to moisture stressed conditions. Hence, these two loci control yield performances under different moisture conditions. The combination of major alleles at all QTL explained variation in all non-moisture stressed environments, and only in a few stressed ones (Supplementary Fig. S4).

To better assess the interaction between QTL, the ‘discovery panel’ entries assigned to the five WP classes were investigated for their haplotype composition at these three QTL (Q.ICD.08, Q.ICD.11, and Q.ICD.44). Four haplotype groups could be identified (Fig. 5). Haplotype 1 with favourable alleles at all loci reached the highest average WP of 6.5 kg ha^-1^ mm^-1^, with 40% of the genotypes belonging to class 2 ‘Responsive to low moisture’. Interestingly, the same allelic combination was responsible for high grain yield under drought (Supplementary Fig. S3). Haplotypes 2 and 3 reached an average WP of 5.3 and 5.4 kg ha^–1^ mm^–1^, respectively, with only one positive allele. Haplotype 2 contributed equally to the four classes, while haplotype 3 was mainly identified in class 4 ‘highly water responsive’. Haplotype 4 harbouring three negative alleles reached 5 kg ha^–1^ mm^–1^, with classes 3 and 4 ‘responsive to high moisture’ and ‘highly water responsive’ corresponding to the highest portions of this haplotype.

### Confirmation of haplotype effect

**Fig. 6.**
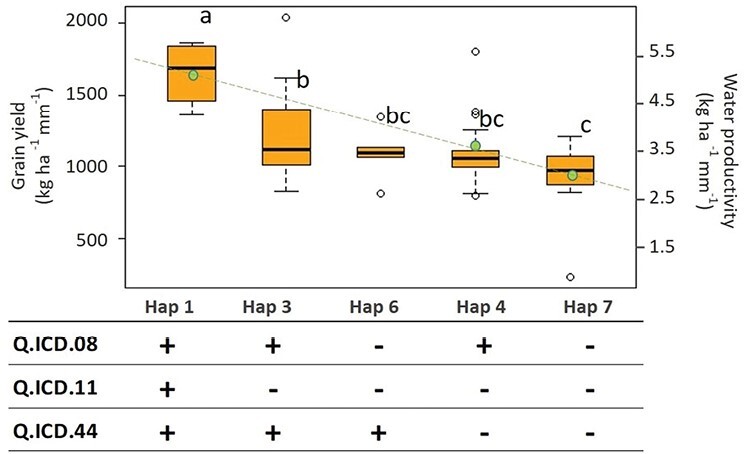
Allelic effect for the combination of three loci associated with GY on the ‘confirmation panel’ under moisture stress. The black line inside the boxes indicates the median of each haplotype across each cluster. ‘+’ mark the positive and ‘-’ mark the negative alleles. Letters (a, b, c) indicate the LSD test. The green dots and dashed line represent the average WP.

Three main QTL (Q.ICD.08, Q.ICD.11, and Q.ICD.44) were investigated for their additive effect within the ‘confirmation panel’. A total of five haplotypes were identified within the panel for these three loci (Fig. 6). The panel was tested under moisture stress in two environments (Sidi el Aydi and Marchouch) during the season 2018–19. The linear model confirmed that the haplotype groups represented discrete classes with significant differences. Haplotype 1 (Hap1) carrying only favourable alleles at all QTL showed a GY advantage of more 705 kg ha^–1^ compared with haplotype 7 with no positive alleles at the three loci and a consequent gain in WP of 2.2 kg ha^–1^ mm^–1^. Also, Hap3, with two positive alleles, except for Q.ICD.11 was significantly superior to Hap7 (no positive alleles), but it was not superior to Hap 4 (only one positive allele). This suggests that Q.ICD.11 had the strongest effect, followed in order by Q.ICD.08 and Q.ICD.44.

### Conversion and validation to KASP

**Fig. 7.**
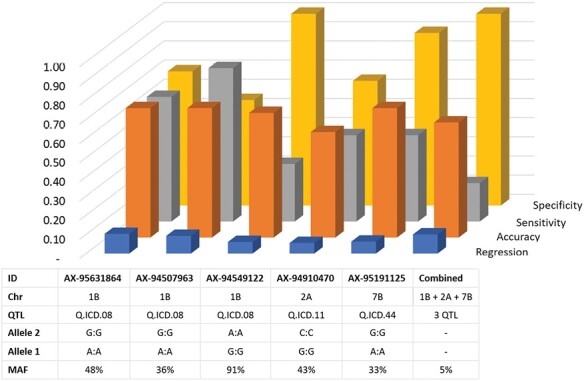
KASP marker validation on an independent set of 94 ICARDA elite lines tested under severe drought. Correlation was measured between the BLUE for grain yield recorded at Sidi el Aydi and the haplotype score. Accuracy, sensitivity, and specificity were determined using only the top 20 and worst 20 yielding lines. AX-95631864, AX-94507963, and AX-94549122 tag Q.ICD-08, AX-94910470 tags Q.ICD.11, and AX-95191125 tags Q.ICD.44.

Marker conversion and validation are quintessential steps to convert the discovery of QTL into usable tools for breeders. Out of 36 array probes known to span the three major QTL (Q.ICD.08, Q.ICD.11, and Q.ICD.44), KASP primers could be designed for 32 of them; of these 17 were purchased and used to screen the ‘validation panel’. Nine of these detected a polymorphism within this elite set (minor allele frequency, MAF >3%). Two explained a significant (*P*>0.05) and three a highly significant (*P*<0.01) portion of the phenotypic variation for grain yield (Fig. 7), when assessing the panel at the severely drought affected station of Sidi el Aydi during season 2019–20. KASP were validated for Q.ICD.08 located on chromosome 1B, and one each for Q.ICD.11 and Q.ICD.44 on chromosomes 2A and 7B, respectively. All five markers were suitable for use in marker assisted selection (MAS), and their use in combination would further increase their independent scores. In fact, AX-95631864 (Q.ICD.08) had the best average performance overall for all criteria, and the highest prediction of phenotypic variation (r^2^=0.10), whereas AX-94507963 (Q.ICD.08) was particularly suitable to identify the top yielders (true positive) with the highest overall sensitivity but with low precision, instead AX-94549122 (Q.ICD.08) and AX-95191125 (Q.ICD.44) have perfect precision (true negative) in identifying the lines to be discarded, but low sensitivity. AX-94910470 tagged the hypothetically strongest QTL (Q.ICD.11) but within the ‘validation panel’ its contribution was minor. However, the combined selection for carrying the positive allele at all five markers resulted in a drastic increase in precision, with only a few top performing lines being selected.

## Discussion

### Water productivity classes explain the genotype response to moisture stress

This study evaluated a global ‘discovery panel’ in 18 environments. Climatic regression (Supplementary Table S6) confirmed that grain yield variation at these environments was mainly controlled by the moisture availability during the vegetative, flowering, and grain-filling phases. Liliane and Charles (2020) and El Haddad *et al.* (2021) also found that moisture availability during the vegetative stage is a critical climatic factor influencing the response of durum wheat genotypes. In fact, the major negative impact of drought stress on wheat is the reduction in fresh and dry biomass production (Farooq *et al.*, 2009), which affects grain number and grain size (Dickin and Wright, 2008). A common response to cope with drought stress is stomatal closure, since that also alters the photosynthetic rate, plants must constantly adjust stomatal conductance to maintain a balance between sufficient CO_2_ uptake and water loss. Toulotte *et al.* (2022) hypothesized that reduced rates of stomatal conductance and subsequently decreased water loss due to reduced stomatal density allowed the available plant resources to be allocated to seed propagation and aboveground biomass. A previous study (Sokoto and Singh, 2013) revealed that water stress at the vegetative stage significantly reduced spike length and grains per spike. However, water stress at flowering and grain filling significantly reduced 1000 kernel weight, grain yield, and harvest index. In fact, water stress-induced accelerated senescence after anthesis shortens the duration of grain filling by causing premature desiccation of the endosperm and by limiting embryo volume (Westgate, 1994).

Principal component clustering based on the most critical climatic factors allowed us to classify the sites into two mega-environments (Fig. 1): moisture stressed and non-moisture stressed. The effect of moisture was further partitioned by assigning genotypes to five classes of WP (Table 2). The same set of genotypes was tested for yield potential under the two mega-environments as well as yield stability overall. Interestingly, the highly yielding genotypes under moisture stress belonged to class 2 ‘Responsive to low moisture’. While under non-stress, the highly yielding genotypes belonged to class 3 ‘Responsive to high moisture’. Class 4 ‘Highly water responsive’ represented mainly the genotypes highly yielding under both conditions (Fig. 2). Similar to Siahpoosh and Dehghanian (2012), genotypes were significantly different for WP. The highly phenotypic variation was due to the environmental effect. The plant response to water stress varied. The decrease of production can be due to plant defence by reducing stomatal conductance and CO_2_ assimilation rate (Catola *et al.*, 2016). When the plant had high WP under moisture shortage, Stallmann *et al.* (2020) explained this reaction by the increase of the intrinsic plant water use efficiency caused by the stomatal closure, which restricts transpiration before it inhibits photosynthesis.

Correlation analysis (Table 2) was done to determine the main traits contributing to grain yield under drought. Interestingly, grain yield was positively correlated with yield components in moisture stressed environments. These findings were consistent with Kiliç and Tacettin (2010) and Al-Ghzawi *et al.* (2018), who reported that spike per m^2^, grains per spike, and TKW were directly related to grain yield. More recent research has shown that it is possible to increase grain size without a negative effect on grain number (Rivera-Amado *et al.*, 2019). The negative correlation between yield under stress and heading date has frequently been reported (Dodig *et al.*, 2010; Gonzalez-Ribot *et al.*, 2017), indicating that the most precocious genotypes would be desirable in accordance with other reports for Mediterranean environments (Acevedo and Ceccarelli, 1989; Quarrie *et al.*, 1999; Richards *et al.*, 2001).

### Genetic dissection of drought tolerance in durum wheat

Breeding cultivars able to thrive under moisture stressed conditions is challenging, since wide adaptation is hindered by high genotype by environment interaction. Drought tolerance is a complex quantitative trait controlled by an army of loci interacting with the environment. Blanco *et al.* (2011) reported that genomic regions linked with GY are present in all chromosomes, and that the magnitude of their effect varies based on the environment. Eleven loci were identified as responsible for the control of GY in more than one environment in our study (Fig. 4; Supplementary Table S8). Four QTL (Q.ICD.04, Q.ICD.07, Q.ICD.37, Q.ICD.39) were active under non-moisture stressed conditions located on chromosomes 1A, 1B, 6A, and 6B, six QTL (Q.ICD.08, Q.ICD.11, Q.ICD.17, Q.ICD.20, Q.ICD.28, Q.ICD.44) on chromosomes 1B, 2A, 3A, 3B, 4B, and 7B were active under moisture stress, and QTL.ICD.41 and Q.ICD.01 on chromosomes 1A and 7A were common under both conditions. Among these QTL, those located on 1A-1B (Q.ICD.04 and Q.ICD.07), 3A-3B (Q.ICD.17 and Q.ICD.20), and 6A-6B (Q.ICD.37 and Q.ICD.39) controlled similar functions, were located on homoeologous physical positions, and hence could represent homoeologous loci on different genomes. Sukumaran *et al.* (2018), Rahimi *et al.* (2019) and Muhu-Din Ahmed *et al.* (2020) all identified QTL on chromosomes 1A, 6A, and 6B related to GY under non-moisture stressed conditions in durum wheat. Interestingly, most QTL also controlled at least one of the yield components. In particular, Q.ICD.01 was linked to all measured four traits (GY, TKW, SPK, and GpS) and a similar region was already identified by other authors for its importance in wheat for GY under different water regimes (Charmet *et al.*, 2001; Ain *et al.*, 2015; Gupta *et al.*, 2017; Rahimi *et al.*, 2019; Muhu-Din Ahmed *et al.*, 2020; Zandipour *et al.*, 2020), yield stability (Sehgal *et al.*, 2020), TKW (Neumann *et al.*, 2010; Lozada *et al.*, 2017; Ogbonnaya *et al.*, 2017), GpS, and SPK (El Hassouni *et al.*, 2019). Similar to previous studies (Shokat *et al.*, 2020; Xin *et al.*, 2020), TKW was positively correlated with grain yield under both moisture-stressed and non-moisture stressed conditions (Table 2), indicating that plant genotypes having higher TKW under irrigated conditions often have a chance to maintain higher TKW under drought conditions (Shokat *et al.*, 2020). Hence, reducing the losses of TKW will result in higher yield under drought conditions. Previous studies have found QTL for TKW on almost all chromosomes of the wheat genome (McCartney *et al.*, 2005; Williams *et al.*, 2012; Okamoto *et al.*, 2013; Zhang *et al.*, 2015; Cabral *et al.*, 2018; Pradhan *et al.*, 2019), and we found the same except on chromosome 7A. The most consistent loci have been detected on chromosomes 3A and 4B; Q.ICD.18 and Q.ICD.28 can be compared with previous findings by Pinto *et al.* (2010) and Sun *et al.* (2017). Grains per spike is correlated with grain yield under both moisture and non-moisture stressed conditions (Pradhan *et al.*, 2019), preserving high GpS during drought conditions is important in order to keep good yield. We found significant associations of GpS (Q.ICD.07 and Q.ICD.31) with chromosomes 1B and 5A under both conditions, while significant regions for spike per m^2^ (Q.ICD.25 and Q.ICD.46) were mainly located on chromosomes 4B and 7B. For AWAI, four MTA spanned on two QTL (Q.ICD.02 and Q.ICD.13) were detected on chromosomes 1A and 2B. Contrary to the finding of Sehgal *et al.* (2017), both QTL were not associated with GY and were instead linked to TKW and grain per spike. Recently, Sehgal *et al.* (2020) identified haplotype blocks associated with stability index Pi on chromosome 1A, using advanced bread wheat lines under contrasting environments. A role for chromosome 2B in the control of GY stability was previously reported in a large elite panel of wheat (Sehgal *et al.*, 2017) and a winter wheat population (Lozada and Carter, 2020), and our study also confirmed its association.

GY showed a significantly positive correlation with TKW, GpS, and SPK (Table 2), indicating that increased GY under moisture stress resulted from increased yield components. While under non-moisture stress, GY increase is due to the significant relationship with TKW and GpS. Consequently, it is feasible to improve GY by selecting these yield related traits in breeding programmes because of the more accurate measurement across moisture and non-moisture stressed environments in comparison with yield.

The three QTL Q.ICD.08, Q.ICD.11, and Q.ICD.44 on chromosomes 1B, 2B, and 7B linked to grain yield under low moisture (Fig. 5), were used to investigate the allelic combination responsible for WP. Interestingly, class 2 genotypes had positive alleles for all three loci providing a significant WP advantage of +1.5 kg ha^–1^ mm^–1^ under low moisture environments. Class 4 had one positive allele at AX-94910470 belonging to Q.ICD.11.

The three main QTL were confirmed by a second independent ‘investigation panel’ grown under moisture stress (Fig. 6). The haplotype assessment confirmed that carrying the positive alleles at all loci increased grain yield by +704.6 kg ha^–1^ and WP by 2.2 kg ha^–1^ mm^–1^. Using a large scale GWAS, Juliana *et al.* (2021) reported the highest number of consistent GY associated markers on chromosomes 2A, 6B, 6A, 5B, 1B, and 7B. Similarly, several studies have also reported QTL on chromosome 1B responsible for the control of GY in durum wheat (Roncallo *et al.*, 2017; Xu *et al.*, 2017; Rehman Arif *et al.*, 2020). The effect of Q.ICD.08 under moisture stress was also identified in a study by Juliana *et al.* (2021) on loci controlling GY under moisture-stressed and non-moisture stressed conditions. This can be explained by the findings of Mathew *et al.* (2019) who reported a root and shoot biomass association region on chromosome 1B. Further, Pshenichnikova *et al.* (2021) found 53 QTL associated with physiological and agronomic traits under contrasting water supply. These findings may explain the importance of Q.ICD.11.

Q.ICD.44 was associated with TKW and GY in the combined analysis and in four individual environments experiencing moisture stress. In a recent mapping population study tested across dry environments, Zaïm *et al.* (2020) found a consistent QTL for GY on the same chromosome. Similarly, by using a diverse population of winter wheat, Lozada and Carter (2020) found a site controlling multiple yield traits and trait stability measures on the same chromosome.

Interestingly, within Q.ICD.08, a gene encoding a hydroxyproline-rich glycoprotein (HRGP) from the late embryogenesis abundant (LEA) family has been pinpointed (Yates *et al.*, 2021). This gene plays a crucial role in enhancing a plant’s ability to withstand drought stress (Ali *et al.*, 2020). The HRGP assists in maintaining optimal cellular hydration, protecting against water loss, stabilizing cell structures, and reducing oxidative damage. HRGP up-regulation during drought stress indicates its involvement in stress-responsive pathways (Ringli *et al.*, 2010; Liu *et al.*, 2020). Further, Q.ICD.11 contains key genes related to water stress response, including an ethylene-responsive transcription factor, a dehydration-responsive element binding protein, and an AP2-like ethylene-responsive transcription factor (Yates *et al.*, 2021). These genes play pivotal roles in enhancing the plant’s ability to face drought conditions. The ethylene-responsive transcription factor is involved in regulating stress-related gene expression (Djemal and Khoudi, 2015), whereas the dehydration-responsive element binding protein contributes to water conservation mechanisms (Agarwal *et al.*, 2017; Buffagni *et al.*, 2020). The AP2-like ethylene-responsive transcription factor aids in orchestrating various stress responses (Chen *et al.*, 2022; Yu *et al.*, 2022). Q.ICD.44 contains genes encoding aquaporin-like proteins (Yates *et al.*, 2021), essential for regulating water movement within the plant. These proteins facilitate efficient water uptake and distribution, aiding a plant in coping with water scarcity during drought conditions (Ayadi *et al.*, 2019).

### Marker validation for marker assisted selection

Axiom to KASP marker conversion and validation was conducted for 17 MTA. Only five KASP markers generated polymorphic haplotypes in the independent set of ICARDA elites IDON43. All five demonstrated significant (*P*<0.05) correlation to grain yield assessed at the severely drought affected station of Sidi el Aydi (Fig. 7). Markers AX-95631864, AX-94507963, and AX-94549122 tag Q.ICD.08 located on chromosome 1B, one of the main loci identified in this study. The first two markers revealed good correlation, accuracy, precision, and sensitivity, whereas the third had medium sensitivity. Similarly, AX-94910470 and AX-95191125 tag Q.ICD.11 and Q.ICD.44, respectively, on chromosomes 4B and 7B. Those markers were protected against Type I errors, but they were prone to Type II errors, with several lines identified as not carrying the positive allele while instead being tolerant to drought. The five markers could be considered as validated and useful for wheat breeding.

## Conclusion

Drought tolerance is a complex quantitative trait that is influenced by genetic background and highly hindered by genotype by environment interactions. To understand the mechanism and the implied loci, a panel was tested under 18 environments, clustered as moisture stressed and non-moisture stressed environments. Our results confirmed that besides grain components, WP is the most critical trait to drive tolerance to moisture stress, and hence should be the primary targets of durum wheat breeders. A total of six QTL were associated with GY under drought, some of them were linked with TKW, GpS, and SPK. The haplotype diversity of three markers each from the three most promising QTL (Q.ICD.08, Q.ICD.11, and Q.ICD.44) sized 19, 83, and 20 Mbp, respectively, caused WP of up to +1.5 kg ha^–1^ mm^–1^ across moisture stressed conditions. Five markers were validated into KASP markers and may further be utilized in MAS. In addition, the remaining QTL might also prove useful after validation into an easier assay to help improve the drought tolerance and yield stability in wheat. The genotypes GID: 800032178, GID: 800030179, and GID: 800043267 were confirmed to be drought tolerant and carrying the positive alleles of the three main QTL. Those genotypes may serve as ideal crossing materials in breeding programmes.

## Supplementary data

The following supplementary data are available at *JXB* online.

Fig. S1. Genome-wide average linkage disequilibrium (LD) decay over genetic distances of the discovery set.

Fig. S2. Boxplots of grain yield (GY) performance and its components across environments.

Fig. S3. Average response of durum wheat water productivity across different rates of moisture.

Fig. S4. Allelic effect for the combination of the three loci associated with GY under moisture stress.

Table S1. Full genotypic and phenotypic datasets for the three germplasm panels used.

Table S2. List of the ‘discovery’ panel and its kinship assignment (k=10).

Table S3. List of the ‘investigation’ panel IDON42 and its kinship assignment (k=8).

Table S4. List of the ‘validation’ panel IDON43.

Table S5. Analysis of variance for GY, DTH, TKW, SPK, and GpS in 18 environments.

Table S6. Linear regression between grain yield and climatic factors across 18 environments and seasonal timepoints.

Table S7. Moisture classes assignments of the 121 selected genotypes and their water productivity characteristics under stressed and non-moisture stressed conditions.

Table S8. Markers associated with the tested traits under moisture stressed and non-moisture stressed mega-environments.

erad357_suppl_Supplementary_Tables_S1-S8

erad357_suppl_Supplementary_Figures_S1-S4

## Data Availability

All data are provided in the article and its supplementary data published online.

## Abbreviations

AMMI: additive main effect and multiplicative interaction
AWAI: AMMI wide adaptation index
b_i_: slope value
BLUE: best linear unbiased estimates
DTH: days to heading
E: environment
G: genotype
G×E: genotype by environment
GpS: grains per spike, interaction
GWAS: genome wide association study
GY: grain yield
IDON: International Durum Observatory Nursery
KASP: kompetitive allele specific PCR
KFD: Kfardan
MTA: marker trait association
MCH: Marchouch
MKZ: Melk Zhar
MUS: Musghar
PCA: principal component analysis
PCR: polymerase chain reaction
PLH: plant height
SPK: spike density per m^2^
QTL: quantitative trait loci
RGA: root growth angle
SAD: Sidi el Aydi
TER: Terbol
TES: Tessaout
TKW: 1000-kernel weight
WP: water productivity
Z: Zadok’s scale

## References

[CIT0001] Acevedo E , Ceccarelli S. 1989. Role of the physiologist-breeder in a crop breeding program for drought resistance conditions. In: Baker FWG, ed. Drought resistance in cereals. Wallingford: CAB International, 117–139.

[CIT0002] Agarwal PK , Gupta K, Lopato S, Agarwal P. 2017. Dehydration responsive element binding transcription factors and their applications for the engineering of stress tolerance. Journal of Experimental Botany 68, 2135–2148. 28419345 10.1093/jxb/erx118

[CIT0003] Ain QU , Rasheed A, Anwar A, Mahmood T, Imtiaz M, Mahmood T, Xia X, He Z, Quraishi UM. 2015. Genome-wide association for grain yield under rainfed conditions in historical wheat cultivars from Pakistan. Frontiers in Plant Science 6, 743. 26442056 10.3389/fpls.2015.00743 PMC4585131

[CIT0004] Al-Ghzawi A , Khalaf Y, Al-Ajlouni Z, Al-Quraan N, Musallam I, Hani N. 2018. The effect of supplemental irrigation on canopy temperature depression, chlorophyll content, and water use efficiency in three wheat (*Triticum aestivum* L. and *T. durum* Desf.) varieties grown in dry regions of Jordan. Agriculture 8, 67.

[CIT0005] Ali M , Gul A, Hasan H, Alipour H, Abbasi AA, Zahra Khan Ft, Abbas S, Fatima T, Taimoor Z. 2020. Chapter 12 - LEA proteins and drought stress in wheat. In: Ozturk M, Gul A, eds. Climate change and food security with emphasis on wheat. Cambridge, MA: Academic Press, 193–205.

[CIT0006] Anyia A , Slaski J, Capo-Chichi L, Chen J, Chang S. 2008. Physiological traits contributing to water productivity and yield stability of barley on the Canadian Prairies. The 5th International Crop Science Congress, Jeju Island, South Korea, 13–18 April 2008.

[CIT0007] Araus JL , Slafer GA, Royo C, Serret MD. 2008. Breeding for yield potential and stress adaptation in cereals. Critical Reviews in Plant Sciences 27, 377–412.

[CIT0008] Ayadi M , Brini F, Masmoudi K. 2019. Overexpression of a wheat aquaporin gene, TdPIP2;1, enhances salt and drought tolerance in transgenic durum wheat cv. Maali. International Journal of Molecular Sciences 20, 2389. 31091755 10.3390/ijms20102389 PMC6566926

[CIT0009] Bassi FM , Brahmi H, Sabraoui A, Amri A, Nsarellah N, Nachit MM, Al-Abdallat A, Chen MS, Lazraq A, El Bouhssini M. 2019. Genetic identification of loci for Hessian fly resistance in durum wheat. Molecular Breeding 39, 24.

[CIT0010] Bassi FM , Sanchez-Garcia M. 2017. Adaptation and stability analysis of ICARDA durum wheat elites across 18 countries. Crop Science 57, 2419–2430.

[CIT0011] Belaid A , Nsarellah N, Laamari A, Nachit M, Amri A. 2005. Assessing the economic impact of durum wheat research in Morocco. Aleppo: International Centre for Agricultural Research in the Dry Areas (ICARDA).

[CIT0012] Bhouri Khila S , Douh B, Mguidiche A, Boujelben A. 2021. Assessing the water productivity of durum wheat in tunisian semi-arid conditions. In: Khebour Allouche F, Abu-hashim M, Negm AM, eds. Agriculture productivity in Tunisia under stressed environment. Cham: Springer International Publishing, 195–211.

[CIT0013] Bilal M , Iqbal I, Rana RM, Rehman SU, Haidery Q-A, Ahmad F, Ijaz A, Umar HMI. 2015. A comprehensive review of effects of water stress and tolerance in wheat (*Triticum aestivum* L.). Tropical Plant Research 2, 271–275.

[CIT0014] Blanco A , Mangini G, Giancaspro A, et al. 2011. Relationships between grain protein content and grain yield components through quantitative trait locus analyses in a recombinant inbred line population derived from two elite durum wheat cultivars. Molecular Breeding 30, 79–92.

[CIT0015] Blum A , Pnuel Y. 1990. Physiological attributes associated with drought resistance of wheat cultivars in a Mediterranean environment. Australian Journal of Agricultural Research 41, 799–810.

[CIT0016] Bradbury PJ , Zhang Z, Kroon DE, Casstevens TM, Ramdoss Y, Buckler ES. 2007. TASSEL: software for association mapping of complex traits in diverse samples. Bioinformatics 23, 2633–2635. 17586829 10.1093/bioinformatics/btm308

[CIT0017] Buffagni V , Vurro F, Janni M, Gullì M, Keller AA, Marmiroli N. 2020. Shaping durum wheat for the future: gene expression analyses and metabolites profiling support the contribution of BCAT genes to drought stress response. Frontiers in Plant Science 11, 891. 32719694 10.3389/fpls.2020.00891 PMC7350509

[CIT0018] Butler DG , Cullis BR, Gilmour AR, Gogel BJ, Thompson R. 2009. ASReml-R reference manual version 4. The State of Queensland, Department of Primary Industries and Fisheries: Brisbane.

[CIT0019] Cabral AL , Jordan MC, Larson G, Somers DJ, Humphreys DG, McCartney CA. 2018. Relationship between QTL for grain shape, grain weight, test weight, milling yield, and plant height in the spring wheat cross RL4452/‘AC Domain’. PLoS One 13, e0190681. 29357369 10.1371/journal.pone.0190681 PMC5777647

[CIT0020] Catola S , Marino G, Emiliani G, Huseynova T, Musayev M, Akparov Z, Maserti BE. 2016. Physiological and metabolomic analysis of *Punica granatum* (L.) under drought stress. Planta 243, 441–449. 26452697 10.1007/s00425-015-2414-1

[CIT0021] Ceccarelli S. 2015. Efficiency of plant breeding. Crop Science 55, 87–97.

[CIT0022] Charmet G , Robert N, Perretant MR, Gay G, Sourdille P, Groos C, Bernard S, Bernard M. 2001. Marker assisted recurrent selection for cumulating QTLs for bread-making related traits. Euphytica 119, 89–93.

[CIT0023] Chen K , Tang W, Zhou Y, Chen J, Xu Z, Ma R, Dong Y, Ma Y, Chen M. 2022. AP2/ERF transcription factor GmDREB1 confers drought tolerance in transgenic soybean by interacting with GmERFs. Plant Physiology & Biochemistry 170, 287–295. 34933148 10.1016/j.plaphy.2021.12.014

[CIT0024] Collard BCY , Mackill DJ. 2008. Marker-assisted selection: an approach for precision plant breeding in the twenty-first century. Philosophical Transactions of the Royal Society B: Biological Sciences 363, 557–572. 10.1098/rstb.2007.2170 PMC2610170 17715053

[CIT0025] del Moral LFG , Rharrabti Y, Villegas D, Royo C. 2003. Evaluation of grain yield and its components in durum wheat under Mediterranean conditions. Agronomy Journal 95, 266–274.

[CIT0026] De Mendiburu F , Yaseen M. 2020. Agricolae: statistical procedures for agricultural research. R package version 1.4.0.

[CIT0027] Dickin E , Wright D. 2008. The effects of winter waterlogging and summer drought on the growth and yield of winter wheat (*Triticum aestivum* L.). European Journal of Agronomy 28, 234–244.

[CIT0028] Djemal R , Khoudi H. 2015. Isolation and molecular characterization of a novel WIN1/SHN1 ethylene-responsive transcription factor TdSHN1 from durum wheat (*Triticum turgidum*. L. subsp. *durum*). Protoplasma 252, 1461–1473. 25687296 10.1007/s00709-015-0775-8

[CIT0029] Dodig D , Zorić M, Kobiljski B, Šurlan-Momirović G, Quarrie SA. 2010. Assessing drought tolerance and regional patterns of genetic diversity among spring and winter bread wheat using simple sequence repeats and phenotypic data. Crop and Pasture Science 61, 812–824.

[CIT0030] Duggal P , Beaty TH. 2008. Genetic epidemiology of infectious disease. In: Kaslow RA, McNicholl JM, Hill AVS, eds. Genetic susceptibility to infectious diseases, 1st edn. New York: Oxford University Press, 3–17.

[CIT0031] El Haddad N , Sanchez-Garcia M, Visioni A, Jilal A, El Amil R, Sall AT, Lagesse W, Kumar S, Bassi FM. 2021. Crop wild relatives crosses: Multi-location assessment in durum wheat, barley, and lentil. Agronomy 11, 2283.

[CIT0032] El Hassouni K , Belkadi B, Filali-Maltouf A, Tidiane-Sall A, Al-Abdallat A, Nachit M, Bassi FM. 2019. Loci controlling adaptation to heat stress occurring at the reproductive stage in durum wheat. Agronomy 9, 414.

[CIT0033] English M. 1990. Deficit irrigation. I: analytical framework. Journal of Irrigation and Drainage Engineering 116, 399–412.

[CIT0034] Farooq M , Wahid A, Kobayashi N, Fujita D, Basra SMA. 2009. Plant drought stress: effects, mechanisms and management. Agronomy for Sustainable Development 29, 185–212.

[CIT0035] Fiedler JD , Salsman E, Liu Y, et al. 2017. Genome-wide association and prediction of grain and semolina quality traits in durum wheat breeding populations. Plant Genome 10, 3. 10.3835/plantgenome2017.05.0038 29293807

[CIT0036] Geerts S , Raes D. 2009. Deficit irrigation as an on-farm strategy to maximize crop water productivity in dry areas. Agricultural Water Management 96, 1275–1284.

[CIT0037] Gonzalez-Ribot G , Opazo M, Silva P, Acevedo E. 2017. Traits explaining durum wheat (*Triticum turgidum* L. spp. *durum*) yield in dry Chilean Mediterranean environments. Frontiers in Plant Science 8, 1781. 29104578 10.3389/fpls.2017.01781 PMC5654942

[CIT0038] Gupta P , Balyan H, Gahlaut V. 2017. QTL analysis for drought tolerance in wheat: present status and future possibilities. Agronomy 7, 5.

[CIT0039] Gupta P , Kabbaj H, El Hassouni K, Maccaferri M, Sanchez-Garcia M, Tuberosa R, Bassi FM. 2020. Genomic regions associated with the control of flowering time in durum wheat. Plants 9, 1628. 33255147 10.3390/plants9121628 PMC7759329

[CIT0040] Habash DZ , Kehel Z, Nachit M. 2009. Genomic approaches for designing durum wheat ready for climate change with a focus on drought. Journal of Experimental Boutany 60, 2805–2815. 10.1093/jxb/erp211 19584119

[CIT0041] Josse J , Husson F, Lê S. 2008. FactoMineR: an R package for multivariate analysis. Journal of Statistical Software 25, 1–18.

[CIT0042] Juliana P , Singh RP, Poland J, et al. 2021. Elucidating the genetics of grain yield and stress-resilience in bread wheat using a large-scale genome-wide association mapping study with 55,568 lines. Scientific Reports 11, 5254. 33664297 10.1038/s41598-021-84308-4 PMC7933281

[CIT0043] Kabbaj H , Sall AT, Al-Abdallat A, Geleta M, Amri A, Filali-Maltouf A, Belkadi B, Ortiz R, Bassi FM. 2017. Genetic diversity within a global panel of durum wheat (*Triticum durum*) landraces and modern germplasm reveals the history of alleles exchange. Frontiers in Plant Science 8, 1277. 28769970 10.3389/fpls.2017.01277 PMC5513985

[CIT0044] Karam F , Kabalan R, Breidi J, Rouphael Y, Oweis T. 2009. Yield and water-production functions of two durum wheat cultivars grown under different irrigation and nitrogen regimes. Agricultural Water Management 96, 603–615.

[CIT0045] Karrou M , Oweis T. 2012. Water and land productivities of wheat and food legumes with deficit supplemental irrigation in a Mediterranean environment. Agricultural Water Management 107, 94–103.

[CIT0046] Kiliç H , Tacettin Y. 2010. The effect of drought stress on grain yield, yield components and some quality traits of durum wheat (*Triticum turgidum* ssp. durum) cultivars. Notulae Botanicae Horti Agrobotanici Cluj-Napoca 38, 164–170.

[CIT0047] Li Y-F , Wu Y, Hernandez-Espinosa N, Peña RJ. 2013. Heat and drought stress on durum wheat: responses of genotypes, yield, and quality parameters. Journal of Cereal Science 57, 398–404.

[CIT0048] Liliane TN , Charles MS. 2020. Factors affecting yield of crops. In: Amanullah, ed. Agronomy: Climate change & food security. Rijeka: IntechOpen.

[CIT0049] Lilley JM , Kirkegaard JA. 2016. Farming system context drives the value of deep wheat roots in semi-arid environments. Journal of Experimental Botany 67, 3665–3681. 26976814 10.1093/jxb/erw093 PMC4896360

[CIT0050] Liu X , McKenna S, Welch LR, Showalter AM. 2020. Bioinformatic identification of plant hydroxyproline-rich glycoproteins. In: Popper ZA, ed. The plant cell wall: methods and protocols. New York: Springer New York, 463––481. 10.1007/978-1-0716-0621-6_26 32617951

[CIT0051] Lozada D , Carter A. 2020. Insights into the genetic architecture of phenotypic stability traits in winter wheat. Agronomy 10, 368.

[CIT0052] Lozada DN , Mason RE, Babar MA, et al. 2017. Association mapping reveals loci associated with multiple traits that affect grain yield and adaptation in soft winter wheat. Euphytica 213, 222.

[CIT0053] Maccaferri M , El-Feki W, Nazemi G, Salvi S, Canè MA, Colalongo MC, Stefanelli S, Tuberosa R. 2016. Prioritizing quantitative trait loci for root system architecture in tetraploid wheat. Journal of Experimental Botany 67, 1161–1178. 26880749 10.1093/jxb/erw039 PMC4753857

[CIT0054] Maccaferri M , Harris NS, Twardziok SO, et al. 2019. Durum wheat genome highlights past domestication signatures and future improvement targets. Nature Genetics 51, 885–895. 30962619 10.1038/s41588-019-0381-3

[CIT0055] Maccaferri M , Sanguineti MC, Demontis A, et al. 2011. Association mapping in durum wheat grown across a broad range of water regimes. Journal of Experimental Botany 62, 409–438. 21041372 10.1093/jxb/erq287

[CIT0056] Malosetti M , Ribaut JM, van Eeuwijk FA. 2013. The statistical analysis of multi-environment data: modeling genotype-by-environment interaction and its genetic basis. Frontiers in Physiology 4, 44. 23487515 10.3389/fphys.2013.00044 PMC3594989

[CIT0057] Mangini G , Gadaleta A, Colasuonno P, et al. 2018. Genetic dissection of the relationships between grain yield components by genome-wide association mapping in a collection of tetraploid wheats. PLoS One 13, e0190162. 29324803 10.1371/journal.pone.0190162 PMC5764242

[CIT0058] Mathew I , Shimelis H, Shayanowako AIT, Laing M, Chaplot V. 2019. Genome-wide association study of drought tolerance and biomass allocation in wheat. PLoS One 14, e0225383. 31800595 10.1371/journal.pone.0225383 PMC6892492

[CIT0059] McCartney CA , Somers DJ, Humphreys DG, Lukow O, Ames N, Noll J, Cloutier S, McCallum BD. 2005. Mapping quantitative trait loci controlling agronomic traits in the spring wheat cross RL4452x‘AC Domain’. Genome 48, 870–883. 10.1139/g05-055 16391693

[CIT0060] Mohammadi M , Blake TK, Budde AD, Chao S, Hayes PM, Horsley RD, Obert DE, Ullrich SE, Smith KP. 2015. A genome-wide association study of malting quality across eight U.S. barley breeding programs. Theoretical and Applied Genetics 128, 705–721. 25666272 10.1007/s00122-015-2465-5

[CIT0061] Mohammadi A , Rafiee S, Mohtasebi SS, Mousavi Avval SH, Rafiee H. 2011. Energy efficiency improvement and input cost saving in kiwifruit production using Data Envelopment Analysis approach. Renewable Energy 36, 2573–2579.

[CIT0062] Mohammadi R , Etminan A, Shoshtari LIA. 2019. Agro-physiological characterization of durum wheat genotypes under drought conditions. Experimental Agriculture 55, 484–499.

[CIT0063] Muhu-Din Ahmed HG , Sajjad M, Zeng Y, Iqbal M, Habibullah Khan S, Ullah A, Nadeem Akhtar M. 2020. Genome-wide association mapping through 90K SNP array for quality and yield attributes in bread wheat against water-deficit conditions. Agriculture 10, 392.

[CIT0064] Neumann K , Kobiljski B, Denčić S, Varshney RK, Börner A. 2010. Genome-wide association mapping: a case study in bread wheat (*Triticum aestivum* L.). Molecular Breeding 27, 37–58.

[CIT0065] Ogbonnaya FC , Rasheed A, Okechukwu EC, Jighly A, Makdis F, Wuletaw T, Hagras A, Uguru MI, Agbo CU. 2017. Genome-wide association study for agronomic and physiological traits in spring wheat evaluated in a range of heat prone environments. Theoretical and Applied Genetics 130, 1819–1835. 28577083 10.1007/s00122-017-2927-z

[CIT0066] Okamoto Y , Nguyen AT, Yoshioka M, Iehisa JC, Takumi S. 2013. Identification of quantitative trait loci controlling grain size and shape in the D genome of synthetic hexaploid wheat lines. Breeding Science 63, 423–429. 24399915 10.1270/jsbbs.63.423 PMC3859354

[CIT0067] Pacheco Á , Vargas M, Alvarado G, Rodríguez F, Crossa J, Burgueño J. 2015. GEA-R (Genotype × Environment Analysis with R for Windows) Version 4.1. CIMMYT Research Data & Software Repository Network.

[CIT0068] Pearson K. 1895. Notes on regression and inheritance in the case of two parents. Proceedings of the Royal Society of London 58, 240–242.

[CIT0069] Pinto RS , Reynolds MP, Mathews KL, McIntyre CL, Olivares-Villegas JJ, Chapman SC. 2010. Heat and drought adaptive QTL in a wheat population designed to minimize confounding agronomic effects. Theoretical and Applied Genetics 121, 1001–1021. 20523964 10.1007/s00122-010-1351-4 PMC2938441

[CIT0070] Pradhan S , Babar MA, Robbins K, et al. 2019. Understanding the genetic basis of spike fertility to improve grain number, harvest index, and grain yield in wheat under high temperature stress environments. Frontiers in Plant Science 10, 1481. 31850009 10.3389/fpls.2019.01481 PMC6895025

[CIT0071] Pshenichnikova TA , Osipova SV, Smirnova OG, et al. 2021. Regions of chromosome 2A of bread wheat (*Triticum aestivum* L.) associated with variation in physiological and agronomical traits under contrasting water regimes. Plants 10, 1023. 34065351 10.3390/plants10051023 PMC8161357

[CIT0072] Qi Y , Yu H, Fu Q, Chen Q, Ran J, Yang Z. 2022. Future changes in drought frequency due to changes in the mean and shape of the PDSI probability density function under RCP4.5 scenario. Frontiers in Earth Science 10, 857885.

[CIT0073] Quarrie SA , Stojanović J, Pekić S. 1999. Improving drought resistance in small-grained cereals: a case study, progress and prospects. Plant Growth Regulation 29, 1–21.

[CIT0074] Rahimi Y , Bihamta MR, Taleei A, Alipour H, Ingvarsson PK. 2019. Genome-wide association study of agronomic traits in bread wheat reveals novel putative alleles for future breeding programs. BMC Plant Biology 19, 541. 31805861 10.1186/s12870-019-2165-4 PMC6896361

[CIT0075] Rehman Arif MA , Attaria F, Shokat S, Akram S, Waheed MQ, Arif A, Borner A. 2020. Mapping of QTL associated with yield and yield related traits in durum wheat (*Triticum durum* Desf.) under irrigated and drought conditions. International Journal of Molecular Sciences 21, 2372. 32235556 10.3390/ijms21072372 PMC7177892

[CIT0076] Reynolds M , Manes Y, Izanloo A, Langridge P. 2009. Phenotyping approaches for physiological breeding and gene discovery in wheat. Annals of Applied Biology 155, 309–320.

[CIT0077] Richards RA , Condon AG, Rebetzke GJ. 2001. Traits to improve yield in dry environments. In: Reynolds MP, Ortiz-Monasterio JI, McNab A, eds. Application of physiology in wheat breeding. Mexico: CIMMYT, 88–100.

[CIT0078] Ringli C. 2010. The hydroxyproline-rich glycoprotein domain of the Arabidopsis LRX1 requires Tyr for function but not for insolubilization in the cell wall. The Plant Journal 63, 662–669. 20545889 10.1111/j.1365-313X.2010.04270.x

[CIT0079] Rivera-Amado C , Trujillo-Negrellos E, Molero G, Reynolds MP, Sylvester-Bradley R, Foulkes MJ. 2019. Optimizing dry-matter partitioning for increased spike growth, grain number and harvest index in spring wheat. Field Crops Research 240, 154–167.

[CIT0080] Roncallo PF , Akkiraju PC, Cervigni GL, Echenique VC. 2017. QTL mapping and analysis of epistatic interactions for grain yield and yield-related traits in *Triticum turgidum* L. var. *durum*. Euphytica 213, 277.

[CIT0081] Sehgal D , Autrique E, Singh R, Ellis M, Singh S, Dreisigacker S. 2017. Identification of genomic regions for grain yield and yield stability and their epistatic interactions. Scientific Reports 7, 41578. 28145508 10.1038/srep41578 PMC5286416

[CIT0082] Sehgal D , Rosyara U, Mondal S, Singh R, Poland J, Dreisigacker S. 2020. Incorporating genome-wide association mapping results into genomic prediction models for grain yield and yield stability in CIMMYT spring bread wheat. Frontiers in Plant Science 11, 197. 32194596 10.3389/fpls.2020.00197 PMC7064468

[CIT0083] Shokat S , Sehgal D, Vikram P, Liu F, Singh S. 2020. Molecular markers associated with agro-physiological traits under terminal drought conditions in bread wheat. International Journal of Molecular Sciences 21, 3156. 32365765 10.3390/ijms21093156 PMC7247584

[CIT0084] Shpiler L , Blum A. 1990. Heat tolerance for yield and its components in different wheat cultivars. Euphytica 51, 257–263.

[CIT0085] Siahpoosh MR , Dehghanian E. 2012. Water use efficiency, transpiration efficiency, and uptake efficiency of wheat during drought. Agronomy Journal 104, 1238–1243.

[CIT0086] Sokoto M , Singh A. 2013. Yield and yield components of bread wheat as influenced by water stress, sowing date and cultivar in Sokoto, Sudan Savannah, Nigeria. American Journal of Plant Sciences 4, 122–130.

[CIT0087] Stallmann J , Schweiger R, Pons CAA, Müller C. 2020. Wheat growth, applied water use efficiency and flag leaf metabolome under continuous and pulsed deficit irrigation. Scientific Reports 10, 10112. 32572060 10.1038/s41598-020-66812-1 PMC7308318

[CIT0088] Sukumaran S , Reynolds MP, Sansaloni C. 2018. Genome-wide association analyses identify QTL hotspots for yield and component traits in durum wheat grown under yield potential, drought, and heat stress environments. Frontiers in Plant Science 9, 81. 29467776 10.3389/fpls.2018.00081 PMC5808252

[CIT0089] Sukumaran S , Xiang W, Bean SR, Pedersen JF, Kresovich S, Tuinstra MR, Tesso TT, Hamblin MT, Yu J. 2012. Association mapping for grain quality in a diverse sorghum collection. The Plant Genome 5, 126–135.

[CIT0090] Sun C , Zhang F, Yan X, Zhang X, Dong Z, Cui D, Chen F. 2017. Genome-wide association study for 13 agronomic traits reveals distribution of superior alleles in bread wheat from the Yellow and Huai Valley of China. Plant Biotechnology Journal 15, 953–969. 28055148 10.1111/pbi.12690 PMC5506658

[CIT0091] R Core Team. 2017. R: A language and environment for statistical computing. Vienna: R Foundation for Statistical Computing.

[CIT0092] Tidiane SA , Chiari T, Legesse W, Seid-Ahmed K, Ortiz R, van Ginkel M, Bassi FM. 2019. Durum wheat (*Triticum durum* Desf.): origin, cultivation and potential expansion in Sub-Saharan Africa. Agronomy 9, 263.

[CIT0093] Toulotte JM , Pantazopoulou CK, Sanclemente MA, Voesenek LACJ, Sasidharan R. 2022. Water stress resilient cereal crops: lessons from wild relatives. Journal of Integrative Plant Biology 64, 412–430. 10.1111/jipb.13222 PMC9255596 35029029

[CIT0094] Tramblay Y , Llasat MC, Randin C, Coppola E. 2020. Climate change impacts on water resources in the Mediterranean. Regional Environmental Change 20, 83.

[CIT0095] Tuberosa R. 2012. Phenotyping for drought tolerance of crops in the genomics era. Frontiers in Physiology 3, 347. 23049510 10.3389/fphys.2012.00347 PMC3446691

[CIT0096] Vitale P , Fania F, Esposito S, Pecorella I, Pecchioni N, Palombieri S, Sestili F, Lafiandra D, Taranto F, De Vita P. 2021. QTL analysis of five morpho-physiological traits in bread wheat using two mapping populations derived from common parents. Genes 12, 604. 33923933 10.3390/genes12040604 PMC8074140

[CIT0097] Wang S , Xu S, Chao S, Sun Q, Liu S, Xia G. 2019. A genome-wide association study of highly heritable agronomic traits in durum wheat. Frontiers in Plant Science 10, 919. 31379901 10.3389/fpls.2019.00919 PMC6652809

[CIT0098] Westgate ME. 1994. Water status and development of the maize endosperm and embryo during drought. Crop Science 34, 76–83.

[CIT0099] Williams K , Munkvold J, Sorrells M. 2012. Comparison of digital image analysis using elliptic Fourier descriptors and major dimensions to phenotype seed shape in hexaploid wheat (*Triticum aestivum* L.). Euphytica 190, 99–116.

[CIT0100] Xin F , Zhu T, Wei S, Han Y, Zhao Y, Zhang D, Ma L, Ding Q. 2020. QTL mapping of kernel traits and validation of a major QTL for kernel length-width ratio using SNP and bulked segregant analysis in wheat. Scientific Reports 10, 25. 31913328 10.1038/s41598-019-56979-7 PMC6949281

[CIT0101] Xu Y-F , Li S-S, Li L-H, Ma F-F, Fu X-Y, Shi Z-L, Xu H-X, Ma P-T, An D-G. 2017. QTL mapping for yield and photosynthetic related traits under different water regimes in wheat. Molecular Breeding 37, 34.

[CIT0102] Xynias IN , Mylonas I, Korpetis EG, Ninou E, Tsaballa A, Avdikos ID, Mavromatis AG. 2020. Durum wheat breeding in the Mediterranean region: current status and future prospects. Agronomy 10, 432.

[CIT0103] Yadvinder S , Kukal SS, Jat ML, Sidhu HS. 2014. Chapter Four - improving water productivity of wheat-based cropping systems in South Asia for sustained productivity. In: Sparks D, ed. Advances in agronomy, Vol. 127. Cambridge, MA: Academic Press, 157–258.

[CIT0104] Yates A D , Allen J, Amode RM, et al. 2021. Ensembl Genomes 2022: an expanding genome resource for non-vertebrates. Nucleic Acids Research 50, D996–D1003. 10.1093/nar/gkab1007 PMC8728113 34791415

[CIT0105] Yu Y , Yu M, Zhang S, Song TA-O, Zhang M, Zhou H, Wang Y, Xiang J, Zhang X. 2022. Transcriptomic identification of wheat AP2/ERF transcription factors and functional characterization of TaERF-6-3A in response to drought and salinity stresses. International Journal of Molecular Sciences 23, 3272. 35328693 10.3390/ijms23063272 PMC8950334

[CIT0106] Yu Y , Ouyang Y, Yao W. 2018. shinyCircos: an R/Shiny application for interactive creation of Circos plot. Bioinformatics 34, 1229–1231. 29186362 10.1093/bioinformatics/btx763

[CIT0107] Zaïm M , Kabbaj H, Kehel Z, Gorjanc G, Filali-Maltouf A, Belkadi B, Nachit MM, Bassi FM. 2020. Combining QTL analysis and genomic predictions for four durum wheat populations under drought conditions. Frontiers in Genetics 11, 316. 32435259 10.3389/fgene.2020.00316 PMC7218065

[CIT0108] Zandipour M , Majidi Hervan E, Azadi A, Khosroshahli M, Etminan A. 2020. A QTL hot spot region on chromosome 1B for nine important traits under terminal drought stress conditions in wheat. Cereal Research Communications 48, 17–24.

[CIT0109] Zhang B , Li F-M, Huang G, Cheng Z-Y, Zhang Y. 2006. Yield performance of spring wheat improved by regulated deficit irrigation in an arid area. Agricultural Water Management 79, 28–42.

[CIT0110] Zhang G , Wang Y, Guo Y, Zhao Y, Kong F, Li S. 2015. Characterization and mapping of QTL on chromosome 2D for grain size and yield traits using a mutant line induced by EMS in wheat. The Crop Journal 3, 135–144.

[CIT0111] Zittis G , Bruggeman A, Lelieveld J. 2021. Revisiting future extreme precipitation trends in the Mediterranean. Weather and Climate Extremes 34, 100380. 34976712 10.1016/j.wace.2021.100380 PMC8686183

