# Supplementary material for: Genomic regions of durum wheat involved in water productivity
Source: J Exp Bot. 2023 Sep 13;75(1):316–33. doi: 10.1093/jxb/erad357 (PMC10735558; doi:10.1093/jxb/erad357)
Supplement: erad357_suppl_Supplementary_Figures_S1-S4 [file erad357_suppl_supplementary_figures_s1-s4.pdf]

# Genomic regions of durum wheat involved in water productivity

Meryem Zaïm, Miguel Sanchez-Garcia, Bouchra Belkadi, Abdelkarim Filali-Maltouf, Ayed Al Abdallat, Zakaria Kehel, and Filippo M. Bassi

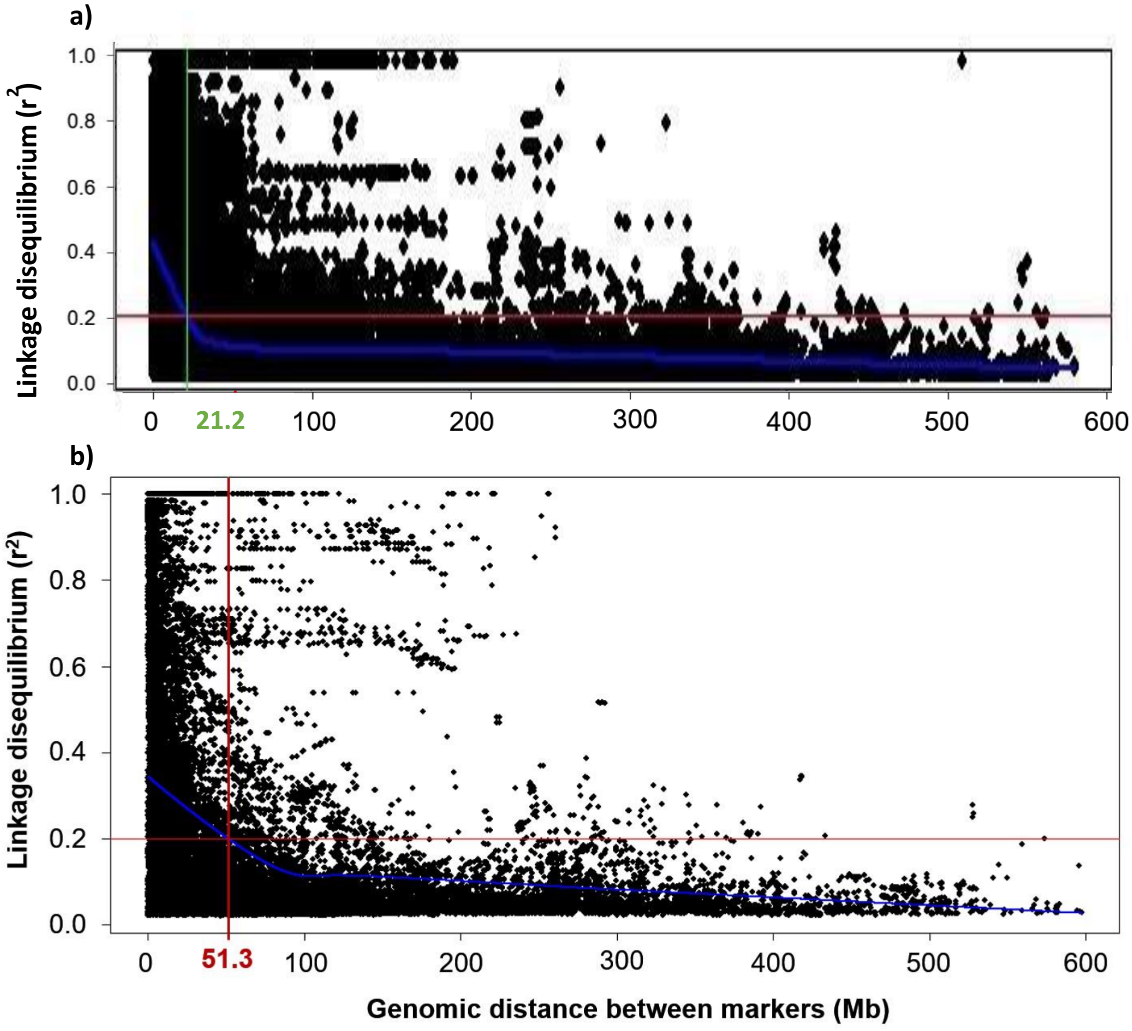


**Figure S1.** Genome-wide average linkage disequilibrium (LD) decay over genetic distances of the second discovery set . Plot of pair-wise LD $r^2$ values as a function of inter-marker map distance (Mbp). The blue curve represents the model fit to LD decay. The red line represents the intercept to $r^2$ = 0.2. a) for the tested panel. b) for the validation set.

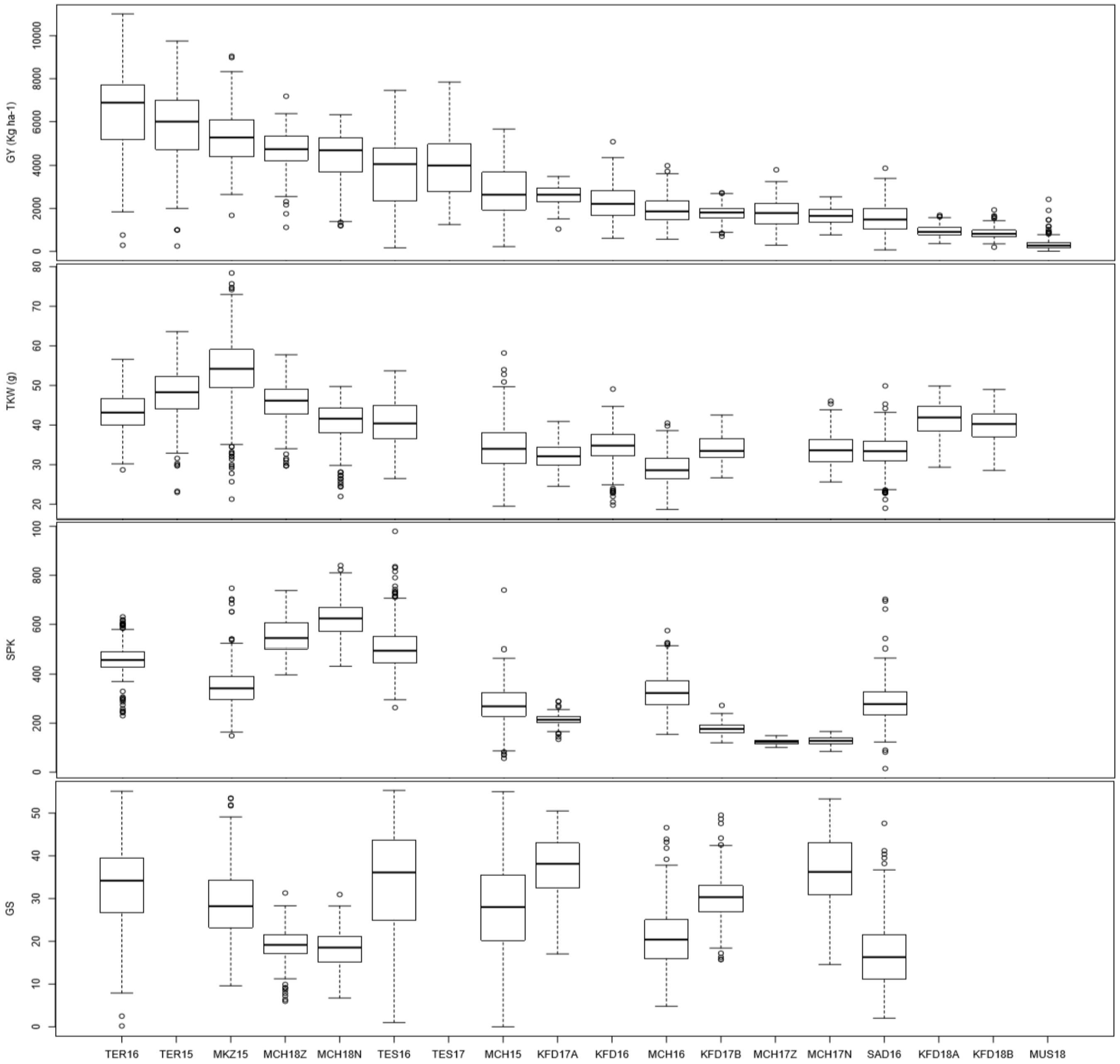


**Figure S2.** Boxplots of grain yield (GY) performances and its components across environments. The medians are indicated by black line inside the boxes. The box borders indicate upper and lower quartiles, the caps indicate 90th and 10th percentiles, and the circles indicate observations below and above those percentiles. TKW: 1,000 kernel weight, SPK: spike density per m2 , GS: grain per spike.

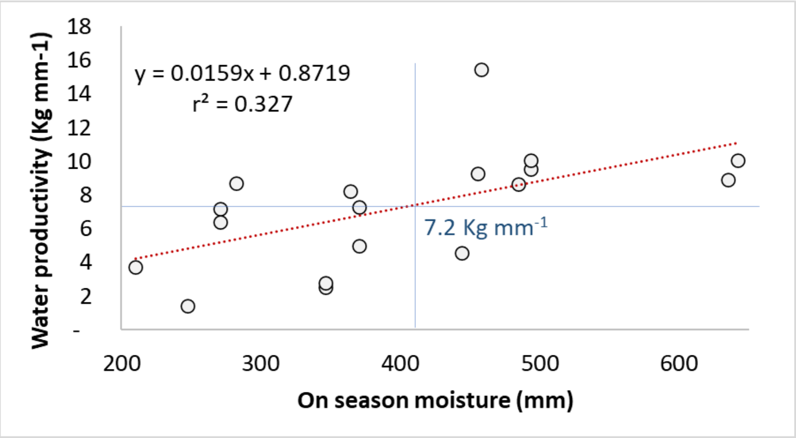


**Figure S3.** Average response of durum wheat water productivity across different rates of moisture.

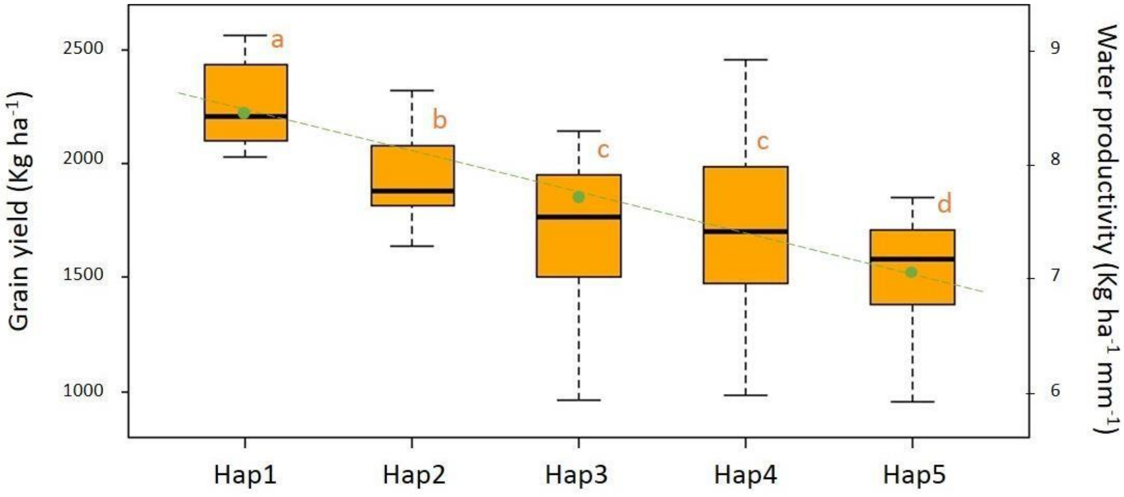


| | | | | | |
|---|---|---|---|---|---|
| QTL.ICD.08 | + | + | + | - | - |
| QTL.ICD.11 | + | + | - | + | - |
| QTL.ICD.44 | + | - | - | - | - |

**Figure S4**. Allelic effect for the combination of the 3 loci associated with GY under moisture stress. The black line inside the boxes indicates the median of each haplotype across each cluster. The "+" for the positive and "-" for the negative alleles. Letters (a, b, c) indicate the LSD test. Green points represent the average water productivity reached by the class "responsive to low moisture" at each haplotype.
